# Integrating Signals from the T-Cell Receptor and the Interleukin-2 Receptor

**DOI:** 10.1371/journal.pcbi.1002121

**Published:** 2011-08-04

**Authors:** Tilo Beyer, Mandy Busse, Kroum Hristov, Slavyana Gurbiel, Michal Smida, Utz-Uwe Haus, Kathrin Ballerstein, Frank Pfeuffer, Robert Weismantel, Burkhart Schraven, Jonathan A. Lindquist

**Affiliations:** 1 Institute of Molecular and Clinical Immunology, Otto-von-Guericke University, Magdeburg, Germany; 2 Institute of Mathematical Optimization, Otto-von-Guericke University, Magdeburg, Germany; 3 Department of Immune Control, Helmholtz Centre for Infection Research, Braunschweig, Germany; Ecole Normale Supérieure, France

## Abstract

T cells orchestrate the adaptive immune response, making them targets for immunotherapy. Although immunosuppressive therapies prevent disease progression, they also leave patients susceptible to opportunistic infections. To identify novel drug targets, we established a logical model describing T-cell receptor (TCR) signaling. However, to have a model that is able to predict new therapeutic approaches, the current drug targets must be included. Therefore, as a next step we generated the interleukin-2 receptor (IL-2R) signaling network and developed a tool to merge logical models. For IL-2R signaling, we show that STAT activation is independent of both Src- and PI3-kinases, while ERK activation depends upon both kinases and additionally requires novel PKCs. In addition, our merged model correctly predicted TCR-induced STAT activation. The combined network also allows information transfer from one receptor to add detail to another, thereby predicting that LAT mediates JNK activation in IL-2R signaling. In summary, the merged model not only enables us to unravel potential cross-talk, but it also suggests new experimental designs and provides a critical step towards designing strategies to reprogram T cells.

## Author Summary

The cells of the mammalian immune system do not exist in isolation, but rather form an integrated network that is constantly scanning the body for signs of ‘foreign’ invasion. Working together, these cells possess the ability to repel invaders and thereby establish protective immunity. One central population in this network are T lymphocytes; whose role it is to coordinate the activity of the adaptive arm of defense. However, T cells constantly receive multiple inputs and therefore it is not clear how they are able to reach a decision. Traditionally, these inputs are studied in isolation using a top-down or stimulus-response approach. Confounding this issue is that our knowledge of these input pathways is not cell-type-specific, but rather represents the sum of all knowledge related to a given stimulus. Therefore, we have undertaken to validate signaling pathways in primary human T cells. We are particularly interested to study the cross-talk between pathways to see how common elements are utilized to make specific decisions that determine cell fate. In doing so, we have identified new components in what were considered to be well-characterized receptor pathways.

## Introduction

A number of receptor signaling networks have been elucidated beginning with the proximal events at the receptor, initiated by ligand binding, and extending down to the level of transcription factor activation. However, this top-down approach to describe pathways usually ignores the potential input coming from other receptor systems. *In vivo*, cells are rarely exposed to only one signal at a time and therefore require the capacity to integrate multiple signals coming from many receptors simultaneously. Thus, a one-to-one relationship between receptor triggering and a functional outcome is usually not possible, as the result of receptor stimulation depends upon the temporal sequence of inputs from multiple receptors. Hence, in order to investigate the cross-talk between receptors, without testing all possible combinations of stimuli, strategies are required to efficiently derive the global signaling network taking advantage of the known isolated top-down pathways of receptors.

A system for which a number of receptors have been characterized and that provides both easy access to material and a short path to medically relevant applications are T cells. T lymphocytes are a central component of the immune system and orchestrate many aspects of the adaptive immune response. This function makes T cells an attractive target for therapeutic intervention, e.g. for treating autoimmune diseases, suppressing immune responses directed against organ transplants, and even in stimulating immune responses against cancer [1]. However, the current immunosuppressive strategies affect all T cells and not only disease-relevant subsets and thus increase the susceptibility to opportunistic infections. In order to circumvent this problem and to identify new targets for potential therapeutic intervention, the interrelationship of the existing signaling machineries must first be understood.

We initially focused our attention on the T-cell receptor for antigen (TCR), which recognizes peptides bound to HLA-molecules. The TCR ultimately determines whether a T cell will become activated or not [2], [3]. However, in order to be able to compare a newly developed strategy with existing therapies, the current drug targets (i.e. receptors or signaling molecules) must first be included in the network. The interleukin-2 receptor (IL-2R) is one such therapeutic target. Activation of T cells via the TCR is known to enhance both the secretion of the autocrine growth factor interleukin-2 (IL-2) as well as the expression of the high affinity form of the IL-2R. On one-hand IL-2 is used to enhance anti-tumor responses or its receptor inhibited in the case of immune suppression [4].

The investigation of cross-talk immediately presents the problem of how to merge signaling networks. Therefore, in order to compare intervention in the TCR signaling pathway with the present therapeutic strategies, we created a tool to generate a merged model that combines our previous logical TCR-signaling network with the IL-2R network.

The IL-2R exists in three forms. The low affinity variant consists of the IL-2Rα-chain (CD25) alone. The intermediate affinity receptor is composed of the IL-2Rβ-chain and the common γ-chain, which is shared with other cytokine receptors. The high affinity form of the IL-2R contains all three chains together and mediates the autocrine feedback-loop [5]. The α- and β-chain mediate ligand binding to initiate signaling via activation of the receptor-associated Janus-kinase 3 (JAK3). Active JAK3 phosphorylates the β-chain of the IL-2R resulting in the recruitment of JAK1 and the adaptor Shc. JAK1 and JAK3 both phosphorylate STAT molecules. Phosphorylated STAT proteins dimerize and translocate to the nucleus. In parallel, Shc recruits Grb2/SoS leading to activation of the RAS-RAF-MEK-ERK cascade. PI3K, JNK, and p38 are also reported to be activated by the IL-2R [5]; however the mechanisms of their activation are not well described.

A number of the key molecules in TCR signaling are also utilized by the IL-2R [5]. The interaction of different pathway modules like ERK and PI3K has been well studied for TCR stimulation. However, the cross-talk with other receptor systems like the IL-2R has rarely been addressed, if at all. It is therefore *a priori* not clear how the common signaling elements of these two pathways interact: can they be cross-activated to enhance signaling, are they used competitively leading to an effective inhibition, or do these modules function independently of one another. Here, our method to merge logical models of signaling networks allows us to identify potential points of receptor cross-talk in a semi-automated manner. To approach a validated version of the signaling network, the merged logical model enables us to design experiments to determine whether potential cross-talks exist or not. Following validation of the IL-2R network in human T-cell blasts, the merged model predicted that STAT signaling should also be initiated upon TCR triggering, which we then verified experimentally. Moreover, our model predicted that LAT should be activated following IL-2 stimulation, which we could verify as well. The ability to reveal new signaling elements in both TCR and IL-2R signaling opens the possibility of gaining new insights into the mechanisms of signaling in T cells that may ultimately identify new targets for T cell-specific therapy.

## Methods

### Ethics statement

Approval for these studies was obtained in writing from the Ethics Committee of the Medical Faculty at the Otto-von-Guericke University, Magdeburg, Germany. Informed consent was obtained in writing in accordance with the Declaration of Helsinki.

### Logical modeling of signaling networks

The simplest model of signaling processes is to collect data on direct molecular interactions in the form of logical formulas, that can be written down in propositional logic [3]: We introduce a logical variable for each signaling component and write down implication formulas for experimentally proven knowledge statements like “*MEK activates ERK*” as *MEK→ERK* and “*STAT3 or STAT5 can induce Blimp-1 expression*” as *STAT3 OR STAT5→Blimp-1*. The precise meaning of the ON-state for the logical variable representing a signaling component depends on the biological context. It can refer to an increase in the amount of a substance (e.g. IP_3_, DAG), its phosphorylation (e.g. LAT), or its recruitment to the membrane or association with other proteins (e.g. Grb2/SoS, PI3K) (the specific meaning for the individual components can be found in Table S1). This interaction network describes the dependencies of activation/inactivation, but does not model the dynamics of these events. The lack of temporal information for the majority of the network components prevents the evaluation of the network using synchronous or asynchronous updates. The propagation of signals would correlate with the number of intermediate steps rather than actual chemical reaction rates. Yet the transient nature of some signals requires at least two networks to properly incorporate all interactions. In particular the discretized versions of negative feedbacks require the ability to represent two mutually exclusive states. As done previously for the TCR network [3], we introduce two time scales for the model: Every implication is assigned a time horizon indicating its validity. Those implications that are only valid for the first time period are called *early implication formulas*, while those valid during the second period are called *late implication formulas*. Implications valid for both time periods are designated *permanent implication formulas*. A permanent implication formula in the TCR network is for example RAS→RAF, whereas CCBLR AND ZAP70→CCBLP1 exemplifies a late implication, thus the dynamics of activation are considered implicitly.

The aim of logical modeling is not to describe the dynamics of a signaling network, but to maintain the interactions rather than when or how. The time horizon allows us to segregate events into discrete steps, which is particularly important in the case of feedbacks. It is clear that the activation of the feedback requires the activity of its preceding signaling elements. The quasi-continuous activity of the signaling components is mapped to discrete states and the ON-state corresponds to full activity. Therefore, there exists a time delay between the detection of the first and full activity of the negative regulator corresponding to the early and late time horizon. Considering transient signaling events the early horizon corresponds to the ascending flank of the signal when the activators dominate and the late horizon to the dominance of negative regulators along the descending flank of the signal. However, since the states of all components are discretized, the state of the logical model is naturally mapped to the peak of the signal (early times) and the adaptation/shutoff of the signaling cascade (late time points).

We assume that in signaling networks a component cannot change its state from active to inactive or vice versa without the influence of either a change of state for other components or external stimulation. For some proteins inactivation may occur via intrinsic mechanisms, e.g. the intrinsic GTPase activity of RAS may result in its inactivation. However, as this activity is far slower than the catalyzed inactivation by GTPase-activating proteins (GAPs), for the purpose of simplification, it is excluded from the model. As introduced previously [6], to model that a component that is not an input to the network (i.e. it has at least one predecessor) can only change its state of activation if there is a reason for it, we introduce the inverse direction of dependency. Therefore we collect all statements with the same right-hand-side leading to activation in one statement *A_1_ OR … OR A_n_ → B* that is transformed to the if-and-only-if (IFF) clause *A_1_ OR … OR A_n _*↔ *B*. This implies that *B* can only be active if at least one of the *A_1_, …, A_n_* is active. We can formalize the standard signaling network in terms of IFF-clauses: Let the IFF-clauses of a given time horizon be denoted as *S_i_* with 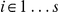. We can then identify the formula 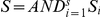 with the network of the biological unit considered: All logical statements *S_i_* with the same time horizon should be valid at the same time to model the global behavior of the unit. Checking these amounts to solving a satisfiability (SAT) problem for the formula S, and each feasible solution represents one possible state of the signaling network. The fact that seemingly simple formulas with AND, OR, and NOT operations are used to represent the information is not a sign of low complexity: In fact, IFFSAT networks are able to encode the NP-complete 3-SAT problem [6], and it is well known that any logical formula can be written in 3-SAT form. In particular, the networks in this paper do not have any of the structures that make solving SAT with some specialized method easier. For more details about the formalism of propositional logic we refer to [7]. With this model at hand, we can then answer relevant biological questions such as predicting the cellular response to a given stimulation using standard solver techniques from computational logic.

An important feature of the logical model is the possibility to detect infeasible subsystems. A typical situation is that following an experiment a subset *K* of components *A_n_* is known to be ON or OFF. If this fixation pattern *K* of components is a possible solution of the network, i.e. every clause *S_i_* can be satisfied, then the fixation pattern is feasible otherwise the fixation pattern *A_n_* is called infeasible. An infeasible pattern, i.e. a conflict within the experimental data, can be due to a modeling error or result from a negative feedback loop that prevents signaling and should only be active at a later time point. The logical network allows us to compute a set of minimal interventions that permits a feasible solution for the fixation pattern *K*. These minimal interventions may represent a solution to the modeling errors, point out inconsistent data in the literature [3], or identify interactions involving negative feedback loops. In the latter case, the interactions are late implication formulas [3]. We note that, as described in [6], negative feedback loops must not necessarily lead to infeasibility of the network and thus, in general, need not be removed, but can be “compensated” by another component. As an example consider the negative feedback loop *PAG* ↔ *CSK, NOT CSK* ↔ *LCKP1, LCKP1* ↔ *FYN, FYN AND NOT TCRB* ↔ *PAG* (Figure S1) occurring in the TCR network. It does not immediately yield an infeasible logical model since TCRB = 1, PAG = 0, CSK = 0, LCKP1 = 1, FYN = 1, is one feasible assignment to the variables. In this case the negative feedback loop is compensated by the additional input TCRB to the IFF-clause producing PAG (Figure S1). Nevertheless, detecting reasons for infeasibility is of major interest, e.g. for monitoring a modeling process or unraveling possible temporal information about interactions. The method described in [6] allows us to efficiently identify all causes of infeasibility and thereby reveals feedback loops as a side result.

### Cell culture and stimulation

Peripheral human T cells were isolated from heparinized blood collected from healthy volunteers using Biocoll (Biochrom AG, Germany) and a Pan T-cell isolation kit II (Miltenyi Biotec, Germany). After resting overnight, T cells were stimulated with plate-immobilized anti-CD3 (OKT3 murine hybridoma, Institute of Molecular and Clinical Immunology, Otto-von-Guericke University, Germany) and anti-CD28 (murine hybridoma 248.23.2, Institute of Molecular and Clinical Immunology, Otto-von-Guericke University, Germany) in culture medium (RPMI-1640 supplemented with 10% heat-inactivated FBS, and 2 µg/ml ciprofloxacin) for 48 hr. After washing twice with RPMI 1640, cells were rested in fresh culture medium for 24 hr.

### Flow cytometry

To determine the number of CD25^+^ cells 1×10^6^ T lymphocytes were washed twice with PBS and incubated with FITC anti-CD25 (BD Biosciences) for 15 min. at 4°C, followed by two additional washing steps. The number of apoptotic cells was determined using an Annexin V/FITC Kit (Bender MedSystems) according to the manufacturer's instruction. Cells were immediately analyzed by flow cytometry (FACS caliber, BD Biosciences).

### Restimulation and inhibition

To identify proteins that became activated upon IL-2R signaling, rested T-cell blasts were stimulated with 100 U/ml IL-2 (Tebu-Bio GmbH, Germany) for the indicated time. To clarify the role of different kinases, T-cell blasts were pre-incubated for 30 min. with either 10 µM PP2, 1 µM wortmannin, 1 µM Gö6976, 1 µM Gö6983 or 1 µM Jak Inhibitor I (Calbiochem, Merck Chemicals, Germany), followed by the addition of IL-2 for the indicated time. Reactions were stopped by addition of ice-cold PBS (Biochrom AG, Germany).

### Immunoblotting

Cells were lysed (1% N-dodecyl β-D-maltoside, 1% NP-40, 1 mM vanadate, 1 mM PMSF, 50 mM Tris pH 7.5, 10 mM NaF, 10 mM EDTA pH 7.5, 165 mM NaCl) for 20 min on ice and centrifuged at 13000 rpm, 10 min, 4°C. The post-nuclear extracts were separated on 10% SDS-PAGE under reducing conditions. Proteins were transferred onto nitrocellulose membranes (Hybond, Amersham Pharmacia Biotech) and immunoblotting was performed using anti-pS473-AKT (93H12), anti-pT202-pY204-ERK1/2, anti-pT180-pY182-p38 MAPK, anti-pT183-pY185-JNK, anti-pY171-LAT, anti-pY705-STAT3 (D3A7), anti-pY694-STAT5 (C11C5); (all Cell Signaling Technology), anti-phosphotyrosine (4G10, murine hybridoma, Institute of Molecular and Clinical Immunology, Otto-von-Guericke University, Germany) and β-actin (Sigma, Germany), followed by incubation with the appropriate secondary HRP-conjugated goat anti-mouse or goat anti-rabbit. Visualization was performed using enhanced chemiluminescence (ECL, Amersham Pharmacia Biotech) according to the manufacturer's instructions. Films were scanned with an Epson Perfection 4990 Photo scanner (Epson America, Torrance, CA).

## Results/Discussion

### Validating the IL-2R signaling network in human T-cell blasts

**Figure 1:**
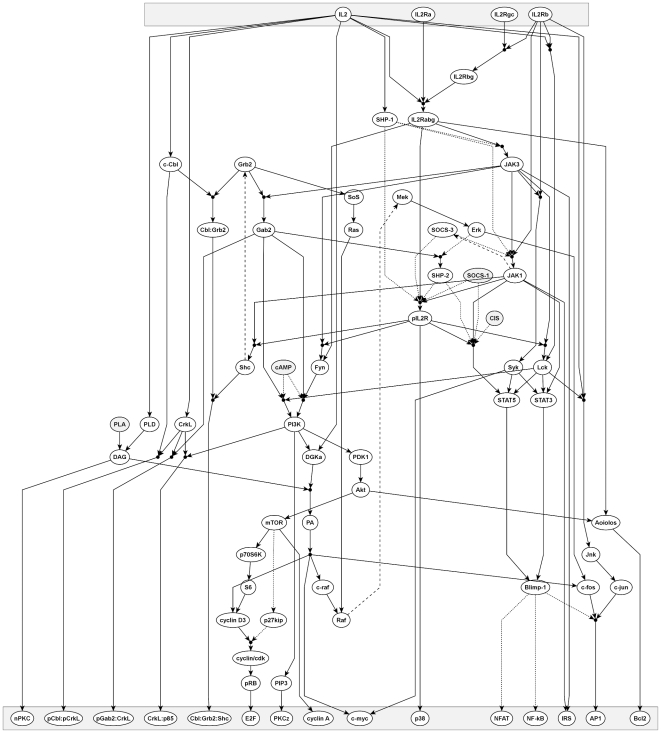
A graphical representation of the logical network of IL-2R signaling. The top layer represents input nodes: the ligand and the receptor subunits. Signaling molecules that are either not regulated by IL-2 signaling, i.e. they can be inputs from other receptors, or for which the corresponding regulation is not known are marked in grey. The bottom layer represents the output, i.e. molecules including transcription factors that become activated upon IL-2R signaling. Solid black arrows indicate activating interactions with a black circle denoting AND-connections. For clarity, activating influences with arrows pointing from the bottom to the top are drawn with dashed black lines. Dotted lines mark inhibitory influences that are expressed as NOT-conditions in the logical network. Detailed descriptions for the interpretation of each node can be found in Table S1.

A number of studies have analyzed the effect of IL-2 on T-cell proliferation, survival, differentiation, and population dynamics [8]–[10]. Our study differs from these as our focus is on the components of IL-2 signaling and their interactions, rather than on the cellular response to IL-2. Starting with the Nature pathway for the IL-2R [11], we generated our own IL-2R signaling network (Figure 1), which contains 68 components and 69 clauses. As done previously for the TCR model [3], only interactions that are reported for IL-2R signaling by at least two independent sources have been included. We favored results generated with untransformed cells, although, due to the limited number of studies and in contrast to the stringency applied to the TCR model, we also considered results that had been generated in T-cell lines.

**Figure 2:**
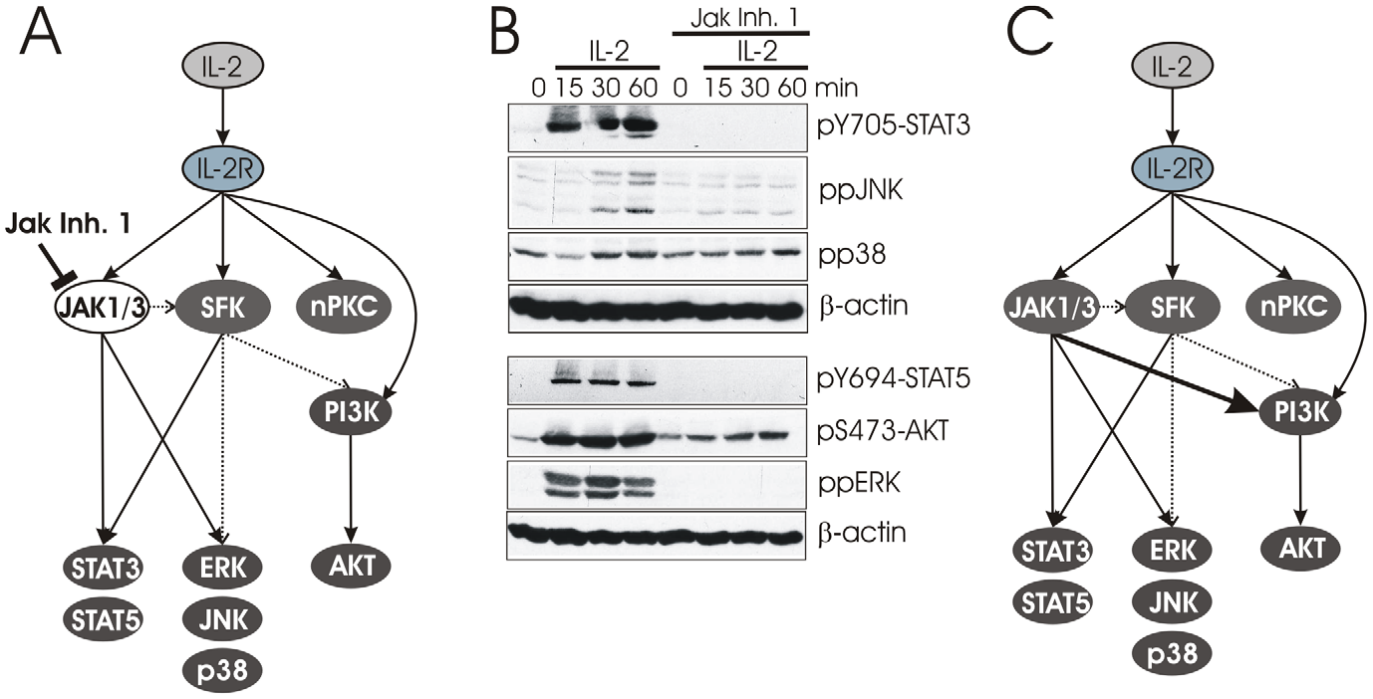
Confirmation of key events of IL-2R signaling in human T-cell blasts. (A) A simplified scheme showing the six major downstream targets identified for IL-2R signaling are grouped into three classes: The PI3K-pathway (AKT), MAP kinases (ERK, JNK, p38), and STAT signaling (STAT3, STAT5). Reliable interactions are indicated with solid lines, potential interactions are presented with dotted lines. The kinases responsible for PI3K activation is not clearly identified and therefore contains the direct information IL-2 activates PI3K (curved line). (B) T-cell blasts were stimulated with IL-2 for the indicated times. Phosphorlyation of AKT, STAT3, STAT5, p38, ERK, and JNK were analyzed by Western blotting. β-actin was analyzed as loading control. (C) Revised model taking into account the strong dependence of PI3K/AKT on JAK1/3 (thick arrow).

The IL-2R network was then validated experimentally using human T-cell blasts. The cells were viable and expressed the high affinity receptor for IL-2 (Figure S2). First, we tested whether all key molecules are indeed activated by the IL-2R upon ligand binding (Figure 2A) thereby targeting the major pathways in the network. Our experiments confirmed the activation of the main downstream targets of the IL-2R: STAT3 and STAT5, the activation of the MAP kinases ERK and JNK, as well as the activation of the PI3K pathway by visualizing phosphorylation of its downstream target AKT. We also found that the pathways of IL-2R signaling show different sensitivities to the dose of IL-2 used. In particular STAT activation is detectable at lower doses than MAPK activation (Figure S3), suggesting different kinase dependencies that may explain the different sensitivities of MAPK and STAT activation. The activation of p38 was not consistently observed over a series of 6 experiments in total (Figure 2B). Furthermore, using Jak Inhibitor I we could demonstrate that all of the target molecules investigated depend on the activation of Janus kinases confirming that JAK3 and JAK1 are the critical kinases immediately downstream of the IL-2R (Figure 2B). The only exception is AKT that still shows some inducible phosphorylation in the presence of Jak Inhibitor I. This implies that at least this pathway depends on a kinase of another family (Figure 2C). However, the strong reduction after inhibition of the JAK kinases demonstrates that the PI3K pathway is largely dependent on JAK1 and/or JAK3, which has not been reported previously.

**Figure 3 pcbi-1002121-g003:**
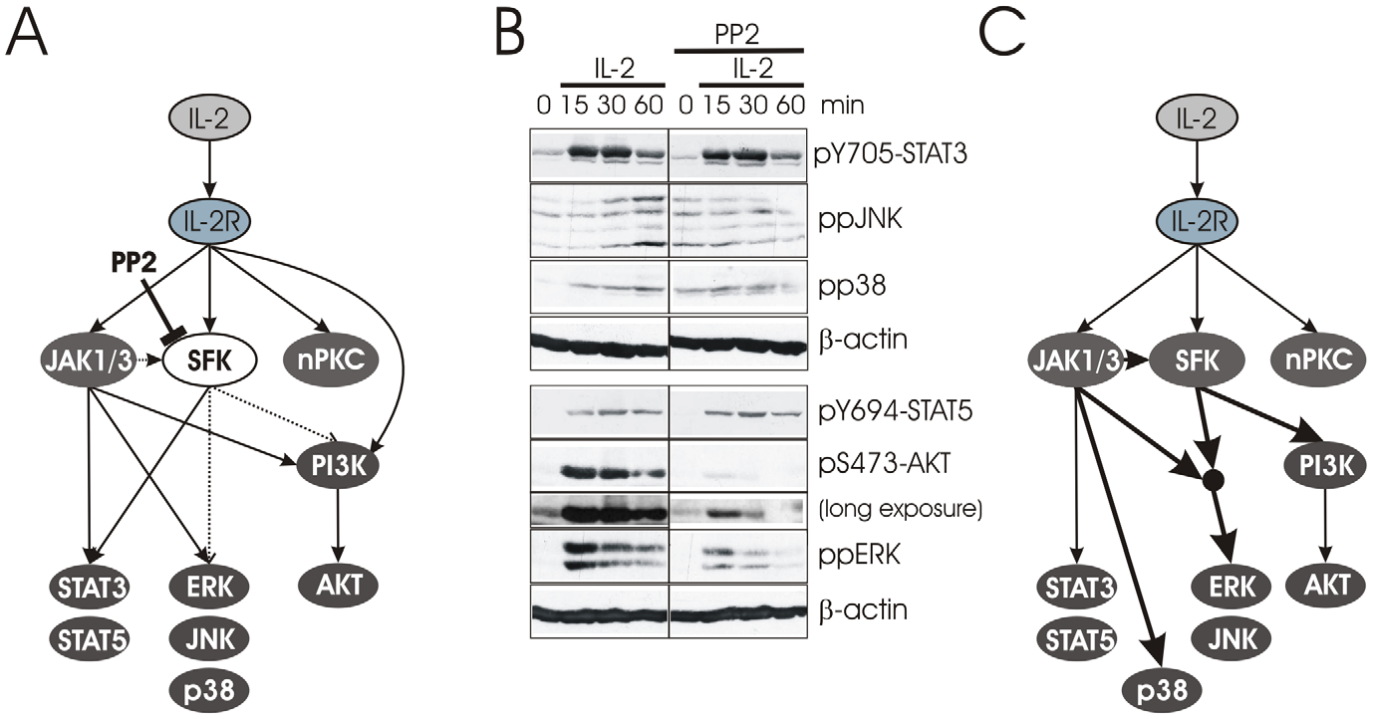
PI3K activation, but not STAT activation is strictly Src family kinase-dependent. (A) Our IL-2R signaling network indicates that only Src family kinases mediate PI3K activation. While STAT activation may be influenced by the Src family kinases Lck and Fyn, Janus kinases alone are sufficient for STAT activation. The model does not contain any information about the kinases-dependence of JNK and p38 activation such that the role of Src family kinases remains unclear (dotted lines). (B) T-cell blasts were stimulated with IL-2 for the indicated times in the presence or absence of the Src family kinases inhibitor PP2. Phosphorlyation of AKT, STAT3, STAT5, p38, JNK, and ERK were analyzed by Western blotting. Note, that the experiment was performed on one gel and irrelevant lanes have been cut out. β-actin was analyzed as loading control. (C) Revised model removing the potential link between SFKs and STATs. PI3K is downstream of SFKs and the dependence of PI3K on JAK1/3 is reconnected to an indirect influence via the effect of JAKs and SFK activation (thick arrows). In addition ERK and JNK are dependent on JAK and SFK while p38 remains independent of SFKs.

One report suggests that PI3K is downstream of a Src family kinase (SFK) in IL-2R signaling [12] (Figure 3A). However, this was the only report that implicates SFKs, although PI3K activity following IL-2 stimulation has been reported multiple times (Table S2). Therefore, to determine whether the data is true for IL-2 stimulation of T cells, we stimulated human T-cell blasts with IL-2 in the presence or absence of the SFK inhibitor PP2 (Figure 3B). We found that AKT phosphorylation is strongly reduced by PP2 treatment. As a positive control, we tested that STAT activation remains normal, since SFK activity is not mandatory. In addition, this experiment suggests that a potential contribution of SFKs to STAT phosphorylation is irrelevant, as the treatment with PP2 had no influence on either STAT3 or STAT5 phosphorylation (Figure 3B) [13]. Therefore the connections between SFKs and STATs were removed. In contrast, the activation of ERK and JNK is dependent on SFKs (Figure 3B) and to our knowledge this has not been shown for IL-2R signaling although the induction of *c-fos* and *c-jun* has been reported to be dependent on Lck [14].

Taken together, the Jak Inhibitor I and PP2 experiments suggest that SFK activity is largely downstream of JAKs (Table S2) since both inhibitors block AKT, but STAT activation is SFK-independent. However, Jak Inhibitor I cannot completely block IL-2-induced AKT activation (Figure 2B). Indeed, one report demonstrated that IL-2R-mediated Lck activity is partially independent of JAK3 [13] and therefore is likely responsible for the weak JAK-independent AKT phosphorylation seen in Figure 2B.

**Figure 4:**
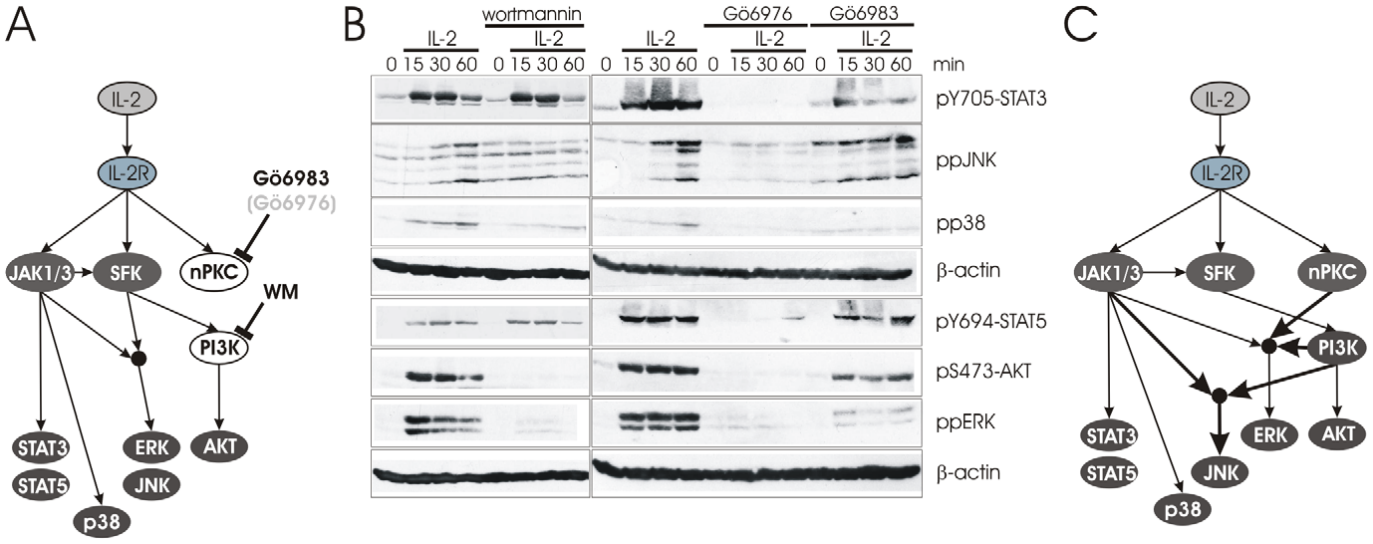
Effects of PI3K and PKC inhibitors on IL-2R signaling. (A) The model predicts that inhibition of PI3K activity should not have any effect STAT or ERK phosphorylation. There exists evidence that PI3K may have an effect on ERK. However, an influence on JNK or p38 is not clear. Although PKCs are activated upon IL-2 stimulation their influence on any other signaling component has not been demonstrated. (B) T-cell blasts were stimulated with IL-2 for the indicated times in the presence or absence of the PI3K inhibitor wortmannin (WM) or the PKC inhibitors Gö6976 and Gö6983. AKT, STAT3, STAT5, p38, JNK, and ERK were analyzed by Western blotting. β-actin was analyzed as loading control. (C) Revised model separating the dependencies of ERK and JNK. ERK depends on JAK1/3, PI3K and nPKCs while JNK is only dependent on JAK1/3 and PI3K (thick arrows).

We next investigated whether PI3K had any influence on other parts of the IL-2R signaling network by applying the PI3K inhibitor wortmannin (WM) (Figure 4A). Figure 4B shows that PI3K does not influence STAT phosphorylation, which is in agreement with our previous result showing that PP2 treatment blocked PI3K activity, but did not influence STAT activation. In contrast, both JNK and ERK are downstream of PI3K (Figure 4B), which fits nicely with the SFK-dependency of these two MAP kinases following IL-2 stimulation (Figure 3B). This result also supports a previous study demonstrating the requirement of PI3K for ERK activation [15]. We noticed that WM and Jak Inhibitor I, but not PP2, are able to completely block ERK activation (Figure 2B, Figure 3B, Figure 4B). Our interpretation of the data is that ERK requires both Janus kinases and PI3K for activation in a non-redundant manner. The discrepancies between PP2 and WM are most likely due to the reversible nature of PP2, such that it is unable to fully inhibit SFKs, resulting in a residual PI3K-activity that appears to be sufficient to support weak ERK activation (Figure 3B). In agreement with this hypothesis, we observe residual AKT phosphorylation after PP2, but not after WM treatment, as WM is an irreversible inhibitor (Figure S4).

Numerous studies performed two decades ago had demonstrated PKC activation upon IL-2R stimulation [16]–[25]. Virtually all of these studies demonstrated an increased membrane-associated PKC activity after IL-2 stimulation using different mouse or human systems. However, there were also conflicting results as to the role of PKCs in IL-2R signaling. While some studies, using PKC inhibitors or phorbol ester-mediated downregulation of PKCs, found that IL-2-induced T-cell proliferation is PKC-dependent [16]–[20] other studies frequently failed to show any effect [21]–[23]. In order to clarify this situation and to determine whether IL-2-induced PKC activation influences the known signaling events in human T-cell blasts, we treated the cells with the PKC inhibitors Gö6976 and Gö6983 (Figure 4B). Gö6976 is an inhibitor of classical PKC isoforms, which depend on calcium, while Gö6983 blocks novel, calcium-independent PKC isoforms. Therefore we expected that Gö6976 would not influence IL-2R signaling, as it is known that calcium is not triggered following IL-2R stimulation [23], [25]. However, to our surprise Gö6976 completely blocked IL-2R signaling. A recent publication identified Gö6976 as an inhibitor of JAK2 of IL-3 signaling in a tumor cell line and, in an additional control, demonstrated that Gö6976 also blocks IL-2R signaling by inhibition of JAK3 [26]. With this in mind, the results of Gö6976 inhibition agree mostly with the results from Jak Inhibitor I by blocking all readouts in our system (Figure 2). Also AKT is now completely blocked unlike in the case of Jak Inhibitor I, which may indicate a reduced specificity of Gö6976 compared to Jak Inhibitor I. Gö6983 was more specific and almost completely blocked ERK activation suggesting that novel PKCs play a role in ERK activation after IL-2 stimulation of human T-cell blasts corresponding to a similar dependency of ERK that was shown for TCR stimulation [27], [28].

In analogy to TCR signaling, ERK depends strongly on SFKs, PI3K, and novel PKCs [3], [27], [28] suggesting a largely common ERK pathway in T cells for both the TCR and IL-2R. It remains an open question where exactly the cross-talk of PKC and PI3K with ERK takes place and whether the pools involved are common between TCR and IL-2R signaling. In other cell systems positive regulation of RAF and MEK by PI3K has been demonstrated [29], [30]. PKCs may also influence ERK activation at the level of RAF by inhibiting the RAF kinase inhibitor protein (RKIP) or by directly phosphorylating RAF itself [31]–[33]. The commonly used signaling components SFKs, PKCs, PI3K, and RAF/MEK/ERK may play a co-stimulatory role in the cross-talk of TCR and IL-2R signaling.

**Figure 5:**
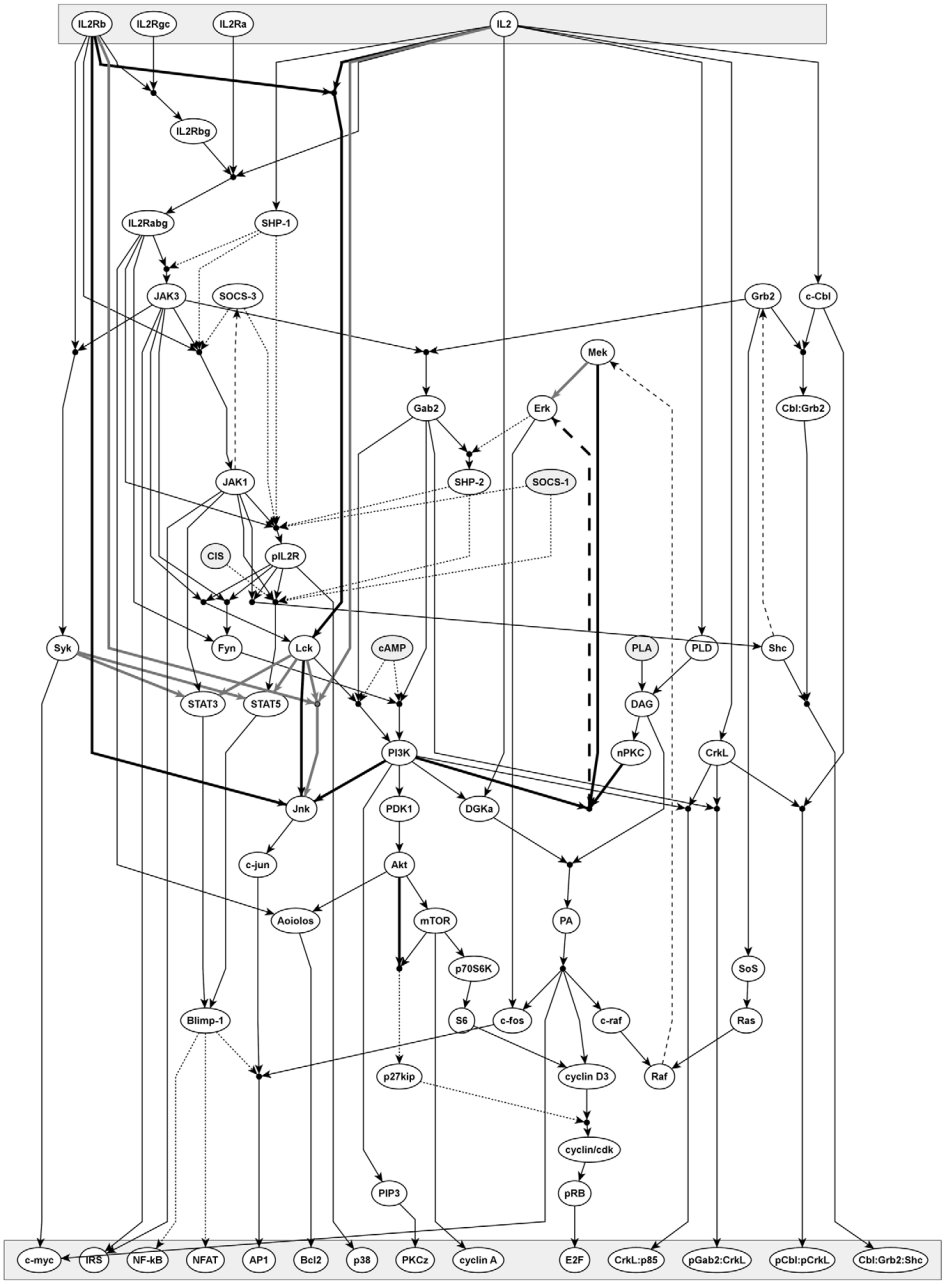
Revised model of IL-2R signaling. The interpretation is similar to Figure 1. The revised connections after the IL-2 experiments in human T blasts are taken into account as indicated in Figs. 2–4 with new connections (including validated potential interactions) shown as thick black lines and removed connections as thick grey lines. The changes are also listed in Table S3. Note, that some connections based on below-quality-standard data remain that are not highlighted (Table S2).

In summary, we present a validated IL-2R signaling network (see Figure 5) containing 68 components and 73 clauses, which was then used for the merging process. The changes to the network are summarized in Table S3.

### Model merging

Cross-talk between two receptors is mediated by molecules that are common to both pathways, but for which the upstream regulation is differentially organized. Thus, in each pathway the regulation of these common molecules is incompletely described from the perspective of a merged network that aims at describing both systems simultaneously. In our example of TCR and IL-2R signaling, synchronous stimulation of both receptors is likely to happen during clonal expansion. Here antigenic stimulation of T cells is still ongoing 30 hours after initial stimulation [34] when the high affinity variant of the IL-2R has been upregulated [35]. Cross-talk may also come into play earlier, as the production of autocrine IL-2 begins as early as 2 hours after stimulation [36]. However the simple “addition” of the regulatory events coming from both receptors may not properly describe their activation in the merged network.

**Figure 6:**
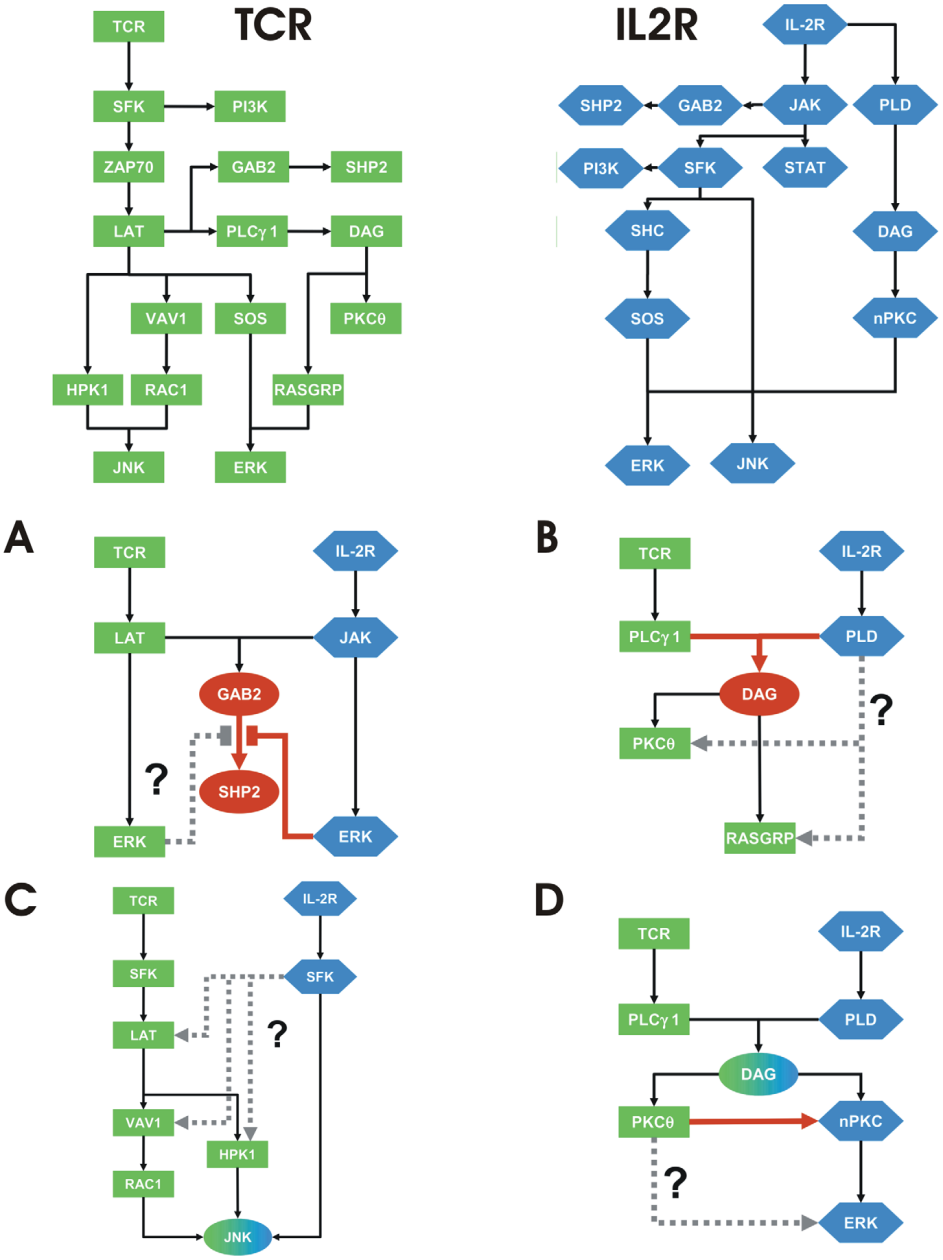
Questions arising during the model merging process. To merge networks, the components of the system require standardization, i.e. components with identical names refer to the same molecule, and the interpretation of their activity states must be consistent. For this example, components colored in green or blue represent the TCR or IL-2R pathways, respectively. During the merging process, the following types of questions appear. In these examples, components highlighted in red represent the focus of a particular question. Grey lines represent potential interactions. A) Implication formulas for the activation of one node have overlapping, but non-identical left-hand-sides, e.g. GAB2→SHP2 versus NOT ERK AND GAB2→SHP2. These clauses differ with respect to ERK. The question is whether ERK always inhibits SHP2 or whether this is a network-specific event involving (unknown) additional regulators that ensure receptor-specificity. B) Two implication formulas for the activation of a node have no overlap. In the simplest case, the mediators are receptor-specific and there is no conflict. If downstream components are activated by one receptor does this mean that these same components are also activated by the other? C) Two implication formulas for the activation of a node have no overlap. It is possible that one is only a simplification of the other that omits intermediate steps. In the IL-2R network JNK is activated directly by SFKs. While the TCR network contains multiple pathways leading to JNK. The first statement is not wrong, since all of these pathways are themselves SFK-dependent. However, the information available is not precise enough to distinguish which pathway is involved. D) The implications formulas depend on different isoforms, e.g. novel PKCs (nPKCs) versus PKCθ which is one member of the nPKCs. Hence, whenever one isoform (PKCθ) is activated, every interaction involving the more general class of proteins (nPKCs) may also be affected and vice versa.

The prerequisites for merging are that the components of the systems are standardized, i.e. components with identical names refer to identical molecules, and the interpretation of their activity states is consistent (see Figure 6). Then the following types of questions appear:

1. Implication formulas for the activation of one node have overlapping, but non-identical left-hand-sides, e.g. *GAB2→SHP2* versus *NOT ERK AND GAB2→SHP2*. These clauses differ with respect to ERK. The question is whether ERK always inhibits SHP2 or whether this is a network-specific event involving (unknown) additional regulators that ensure receptor-specificity (see Figure 6A).
2. Two implication formulas for the activation of a node have no overlap. In the simplest case, the mediators are receptor-specific and there is no conflict. If downstream components are activated by one receptor does this mean that these same components are also activated by the other (see Figure 6B)?
3. Two implication formulas for the activation of a node have no overlap. It is possible that one is only a simplification of the other that omits intermediate steps. In the IL-2R network JNK is activated directly by SFKs. While the TCR network contains multiple pathways leading to JNK. The first statement is not wrong, since all of these pathways are themselves SFK-dependent. However, the information available is not precise enough to distinguish which pathway is involved (see Figure 6C).
4. The implications formulas depend on different isoforms, e.g. novel PKCs (nPKCs) versus PKCè which is one member of the nPKCs. Hence, whenever one isoform (PKCè) is activated, every interaction involving the more general class of proteins (nPKCs) may also be affected and vice versa (see Figure 6D).

This list contains specific examples, however a general problem occurs when the “local interaction” information taken into account while constructing signaling networks is of a different resolution with respect to the proteins involved. The different forms of inherently incomplete information result in obstacles for the merging of two networks, which can be formalized by considering the inverse problem: How can one extract the signaling network or an arbitrary subnetwork of one receptor from a merged network (further discussion on this issue can be found in Text S1).

In contrast to model construction from interaction databases or large-scale association screens, our TCR and IL-2 networks include only causal interactions that have been shown under comparable experimental conditions. This explains the rather sparse network structure, compared to say interactome database models, but sparseness is not a necessary prerequisite for applying the merging process. We believe that such a well-curated model is, applying Occam's Razor, often more helpful in understanding cellular behavior than a model that includes all potential interactions. Clearly, the scalability of the merging process depends on two factors: The first is our ability to compute intersections, unions, and induced subgraphs in labeled directed graphs, which is computationally easy. The second is our ability to solve SAT problems of a size less than or equal to a direct sum of the parent networks. SAT problems are in a mathematical sense computationally hard. There are however good implementations of exact SAT solvers available that routinely handle problems with 200,000 variables and a million clauses. This appears to be sufficient even for human interactome size networks. For the merging process, the graph density or connectivity index of the networks is not as much of importance as the structure of the overlap. As noted, the complexity of computing a logical projection is higher than that of solving an instance of SAT (see Text S1), and in practice the difficulty here depends very much on the amount of non-identical subnetworks that share many components. Manual merging, however, has to perform the same checks, which is always error-prone and quickly becomes impossible even in models of the size of the TCR and IL-2 network presented here. Therefore, any automatic support is useful in the model merging process, since it will always be faster than solving the consistency checks by hand.

In the course of modeling, the question of how to consistently merge two or more models arises in two contexts: First, when two independently developed networks with some common components shall be integrated into one network. And second, when multiple extensions to a base model are to be merged, for example after different parts of the base network have been refined. In the merging algorithm (see Text S2) we call the existing networks ‘parents’, and the merged network ‘child’, hinting at the close relationship with version control of data. The task of merging cannot be completely automatized, unless one is satisfied with annotating each statement with a label stating which network it came from. In this case no overlap actually occurs, but the resulting network is nothing more than a disjoint union of its parents. Assuming compatible data quality many steps can be automated and expert intervention is needed only in those cases where it will lead to decisions about new experiments. We implemented this in our in-house modeling system with about 300 lines of LISP code.

An attractive feature of the merging process of logical networks is the ability to confirm the validity of cross-talk between pathways. Not all possible cross-talks arising from the merging process are feasible, since the activation patterns of each individual pathway must be recovered in the combined network. In a typical situation, a potential realization is that a cross-talk activates or inactivates molecules that contradict the known activation pattern. Moreover, the logical model is not only able to tell that a cross-talk contradicts existing data; it can also offer minimal interventions to correct this inconsistency [6]. In this way, the number of possible cross-talks is reduced and/or modeling errors, which occurred during the initial modeling or following the merging process, can be revealed. In particular it allows us to determine late implication formulas that usually describe negative feedback loops [3] (see Methods). Among the set of identified pools of molecules that mediate cross-talk, the logical model allows us to easily identify potential cross-inhibitions among the pathways by commonly used negative regulators. These may be either new potential cross-talks or can be ruled out by existing data such that they must not be active during initiation of signaling and are therefore molecules that are regulated by late implication formulas. The identification of cross-inhibition is of particular interest when applying sequential stimuli. The model allows us to predict whether the pre-stimulation of one receptor prevents the activation of particular pathways triggered by the second receptor and finally the global outcome of sequential stimulation.

### Possible cross-talk between the TCR and IL-2R

We merged the validated IL-2R-network with our existing TCR-model (Figure 5 and Figure S5 to yield Figure S6). The merged network containing 150 components and 167 clauses (see Table S2). The merging process generated two classes of questions. First, do commonly used signaling proteins form separate pools? If not, is there cross-talk between pathways mediated by these proteins? Second, provided signaling elements are commonly triggered, can missing details for upstream activators in one pathway be elucidated from the other signaling pathway? This second question is similar to asking at which points two signaling pathways feed into a shared signaling module. It also corresponds to the transfer of information from one parent to the other, via the child. By projecting a subnetwork we thus improve the details of classical top-down receptor pathways. In summary, we ended up with the following list of questions that encouraged experimental consideration:

1. *cAMP is an inhibitor of PI3K activation in the IL-2R signaling network*. cAMP is a known inhibitor of Lck and Fyn and this mechanism has recently been shown to work for TCR signaling as well in the context of cross-talk with the μ-opioid receptor [37]–[39]. Since we already demonstrated a SFK-dependency of PI3K activation (Figure 3B) we therefore did not consider it necessary to investigate this question further.
2. *One possible element for cross-talk can be PI3K*. For example the strong PI3K-dependence of ERK activation in both signaling systems may give rise to cross-regulation between TCR and IL-2R signaling mediated by PI3K.
3. *The TCR and the IL-2R use different pathways to generate the second messenger DAG (PLCγ1 and PLD, respectively)*. However, it has been suggested that the different DAG species trigger different sets of effectors [40]. Still, it seems that DAG in both cases activates PKCs [16], [17], [20]–[22], [24], [41] although the downstream signaling component that may explain the pro-proliferative effect of PKC activation in IL-2R signaling had not been identified previously [16], [17], [20]–[23]. We could show that, similar to TCR signaling, ERK activation depends on novel PKCs (Figure 4B) suggesting that the origin of DAG is irrelevant for PKC activation and its effects on ERK. In addition, DAG effectors may be commonly used by the IL-2R and the TCR. The RAS activator RasGRP1 is such an effector that is present in the TCR signaling network, but has been excluded from IL-2R signaling by a recent study [42].
4. *In the IL-2R signaling network the activation of SHP2 by recruitment to the adaptor GAB2 can be prevented by ERK*. Engaging this question experimentally in primary cells has proved challenging due to a lack of suitable reagents directed against GAB2. Thus, it remains an open question whether ERK can modulate SHP2-GAB2 interactions in TCR signaling.
5. *Both receptors activate the SFKs Lck and Fyn*. However, it is not clear whether common pools are used or if cross-activation is possible. Although we have excluded a role for SFKs in the activation of STAT3 and STAT5 by the IL-2R, the possibility that SFKs mediate STAT activation under other conditions still exists [43].
6. *The pathways leading to p38 and JNK activation are known in quite some detail for the TCR network, but not for IL-2R signaling*. This suggests that we experimentally verify whether some, all, or none of these elements are utilized by the IL-2R.

### STAT activation upon TCR stimulation

**Figure 7:**
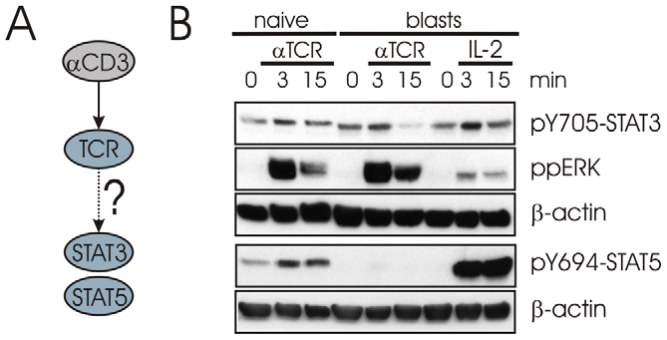
Cross-talk of the TCR and IL-2R signaling network. (A) STAT activation after TCR-stimulation. (B) Naïve T cells were stimulated with cross-linked CD3×CD28 for the indicated time. T-cell blasts were stimulated with IL-2 or re-stimulated with cross-linked CD3×CD28 for the indicated times. The tyrosine phosphorylation of STAT3 and STAT5 was analyzed by Western blotting. ERK activation was analyzed to confirm proper stimulation of the TCR. β-actin was analyzed as loading control.

One report demonstrated that Lck is able to phosphorylate STAT proteins *in vitro* and although being activated after IL-2 stimulation, Lck is not required for IL-2R-mediated STAT activation (see Figure 3B) [13]. Since SFKs can activate STATs under other circumstances [43], we thought to test in the context of TCR signaling whether the phosphorylation of STATs by Lck and/or Fyn may play a role. We therefore looked for the activation of STAT3 and STAT5 after TCR stimulation using cross-linked CD3×CD28 in both primary human T cells and human T-cell blasts. Following TCR-stimulation, both phospho-STAT3 and phospho-STAT5 are weakly induced in naïve T cells, but not in T-cell blasts (Figure 7). A basal level of STAT tyrosine phosphorylation is present in naïve T-cells, but absent in T-cell blasts in the case of STAT5. Moreover it appears that STAT3 tyrosine phosphorylation is lost upon TCR stimulation in human T-cell blasts (Figure 7). Since STATs are downstream of many cytokine receptors involved in homeostatic signaling of T cells, the suppression of STAT3 activation by the TCR may represent a mechanism to switch off certain homeostatic signals upon TCR stimulation. In summary, TCR and IL-2R may cross-talk via a common pool of SFKs; however this question will require further investigation. An alternative possibility could be that STATs are activated by a member of the Syk family of protein tyrosine kinases [13]. The TCR is reported to activate both ZAP-70 and Syk [45], although substrates for Syk in TCR signaling are not well defined. A third option is that TCR activates JAKs directly, however this possibility has been excluded by a previous study [44].

The phosphorylation of both STAT3 and STAT5 following TCR stimulation has previously been reported in T-cell lines [45], [46]. Both studies also demonstrated that STAT activation was dependent on SFKs. In addition, another study demonstrated that JAKs are not induced by TCR stimulation [44]. These studies were not included in our TCR signaling network for two reasons: First, each was reported only once and second, there exist conflicting reports claiming the absence of STAT3 or STAT5 activation upon TCR stimulation in human T cells [44], [46]. Interestingly, our logical modeling approach suggested that the TCR mediates STAT activation, thus we were able to resolve these conflicting reports for the human system and demonstrated for the first time STAT3 activation following TCR stimulation in naïve human T cells. Of the conflicting reports, the study using a human CD4^+^ T-cell line [46] is in agreement with our results for naïve T cells that STAT3 can be activated after TCR stimulation and suggests that the cell line is more naïve T-cell like. Also the inability of TCR stimulation to induce STAT3 activity in human T-cell blasts [44] is in agreement with our results for human T-cell blasts and highlights a difference in TCR signaling in naïve human T cells versus human T-cell blasts. In agreement with our results in naïve human T cells, in the murine system STAT5 is activated after stimulation with cross-linked anti-CD3 or peptide-loaded antigen-presenting cells [45] confirming that the STAT activation takes place under physiologic stimulation conditions. We could also confirm that STAT3 and STAT5 are activated following TCR stimulation in naïve mouse T cells as well as in mouse T-cell blasts (Figure S7). Taken together, the subtle differences in STAT3 and STAT5 activation point towards a rewiring of the signaling networks in activated human T cells that appears to be species-specific as these differences are not observed in mice. A potential role for CIS in mediating the block in TCR-induced STAT activation in T-cell blasts can be excluded, as IL-2R-mediated STAT activation is normal [47]. TCR-mediated STAT activation should support proliferation and cell survival as STATs are known to activate a number of important genes including cyclins as well as members of the Bcl-family [48].

### LAT is phosphorylated following IL-2R stimulation

**Figure 8:**
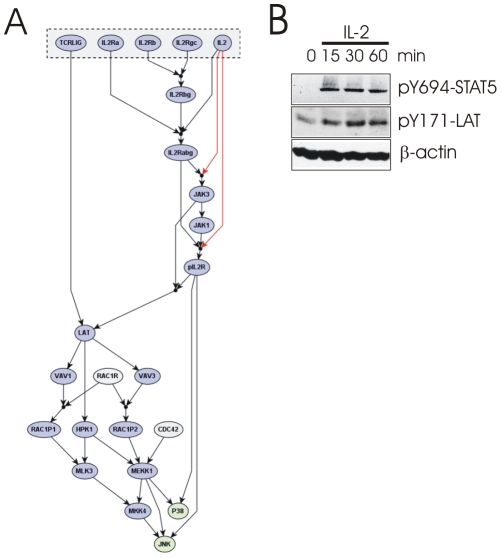
Potential pathways to JNK activation for IL-2R signaling derived from TCR signaling. (A) The TCR signaling network offers two main routes to JNK activation involving the key molecules Rac/Cdc42 and HPK1. A number of upstream events known for TCR signaling may also be triggered by IL-2R signaling. (B) T-cell blasts were stimulated with IL-2 for the indicated times. STAT5 and LAT phosphorylation were analyzed by Western blotting. STAT5 phosphorylation indicates successful IL-2 stimulation. β-actin was analyzed as loading control.

The merging of signaling networks also allows a well-defined information transfer between receptor pathways. The level of detail with respect to the activation of certain pathways is usually different for two receptors. In our networks, this applies in particular to the activation of JNK after IL-2 stimulation. However, merging with the TCR signaling network provided essentially two pathways: Rac/Cdc42 activation or a pathway via HPK1 (Figure 8A). As it is notoriously difficult to show HPK1 activation in primary cells, we looked to see whether LAT is involved in IL-2 mediated JNK activation, as in TCR signaling HPK1 is known to influence JNK activation via the LAT complex [49], [50]. Indeed LAT becomes tyrosine phosphorylated following IL-2 stimulation of human T-cell blasts (Figure 8B). Thus, we have uncovered a known pathway that was previously not described to be involved in IL-2R signaling. Elucidation of this connection will require further investigation, as our TCR network predicts a number of downstream effectors of LAT that may now also be triggered by IL-2. Therefore, we propose that phosphorylation of LAT may be a first indicator to the JNK-activation pathway in IL-2 stimulated human T-cell blasts. An additional consequence is that our model now predicts several completely new signaling branches with respect to IL-2R signaling such as Vav and SLP-76 (SH2 domain containing leukocyte protein of 76 kDa), which may be shared with the TCR and will require further experimental investigation.

A recent study has also revealed a potential role for IL-2 in the homeostatic proliferation of naïve CD8^+^ T cells [49]. Although our network was established for the high affinity receptor, the signaling network should be largely valid as signaling occurs via the cytoplasmic tail of the β- and common γ-chain of the IL-2R ([5] and references in Table S2). Thus, our result that LAT is involved in IL-2R signaling may also apply to naïve T cells. It also correlates nicely with the observation by Cho et al. [51] that the IL-2 response of naïve CD8^+^ T cells depends on the recruitment of the IL-2Rβ-chain into lipid rafts were LAT is localized [52] and our observation of IL-2-induced LAT phosphorylation may constitute the molecular mechanism behind the observations of Cho et al.

**Figure 9:**
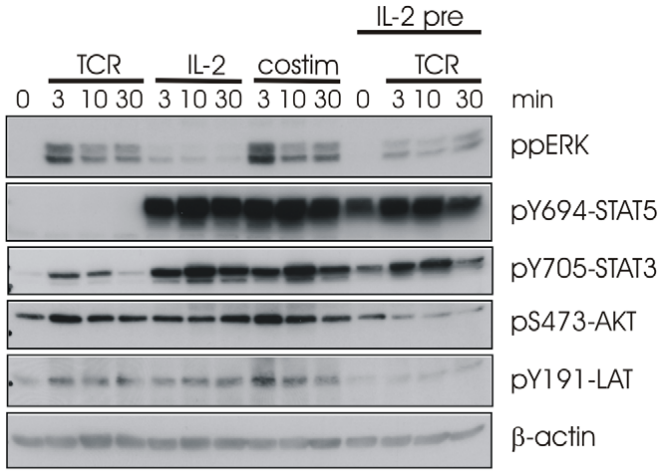
Influence of IL-2 upon TCR signaling. Human T-cell blasts were stimulated with either CD3×CD28 alone, IL-2 alone, both receptors stimulated simultaneously, or the cells pretreated with IL-2 for 30 min before adding CD3×CD28. Following application of the stimuli, samples were taken for analysis at the times indicated. Blots were probed with the antibodies indicated.

The final issue remaining is what influence IL-2 has upon TCR signaling. One could envision that these signals might intersect during clonal expansion. Potential points of intersection are at the level of DAG, SHP1, Lck, and/or PI3K. The first two have the potential for inhibition, whereas the latter may work synergistically. The Boolean nature of the model prevents a reliable prediction of synergistic increase of the activation of a pathway since the component is either ON or OFF and there exists no state with higher activity than ON. We can however calculate the effect of IL-2 pre-stimulation on subsequent TCR signaling. We opted for this combination of stimulation as it is well known that T cells down-regulate TCR expression following activation. Additionally, we know from our previous work [53] that autocrine IL-2 does not prevent sustained TCR signaling. Taking into account that with IL-2 prestimulation the TCR stimulation occurs when IL-2R signaling is already in its late phase, the Boolean network predicts that ERK and AKT remain inactive after stimulation of the TCR (Table S3). To address this question experimentally we stimulated human T-cell blasts with either CD3×CD28 alone, IL-2 alone, both receptors simultaneously, or pre-treated the cells for 30 min with IL-2 before adding CD3×CD28 (Figure 9). As a control, the level of receptor surface expression was monitored to ensure that IL-2 pre-treatment did not alter the level of surface TCR (see Figure S8). From the data presented, it appears that costimulation of both receptor systems has an additive effect, potentially via Lck and/or PI3K. However, due to the discretization of the model, such effects are not represented. Here, molecules are simple active or not and changes in the level of activity are therefore not described. In contrast, pre-incubation with IL-2 appears to result in a desensitization of the cells towards TCR stimulation suggesting activation of negative regulators, such as SHP1. The inhibitory effect is most striking for ERK, AKT, and LAT, which are predominately utilized by the TCR. We therefore concluded that the temporal application of stimuli is critical for interpreting the consequence of cross-talk.

Following T-cell activation, the activated T-cell clone rapidly expands into an effector population. As the number of T cells outnumbers the APC one could envision that a period of desensitization towards further TCR signaling might benefit the immune response by preventing the TCR-induced stop signal [54]. Thus allowing activated T cells to escape the lymph node and migrate into the periphery.

### Conclusion

In summary, our results demonstrate the importance of investigating receptor cross-talk and show that logical modeling is indeed an appropriate method to address this topic. The TCR and the IL-2R are two receptors for which the signaling events are known in great detail. However, our investigation of the merged receptor networks has allowed us to uncover previously unknown events in both signaling pathways as well as to identify points of intersection. An improved understanding of the molecular interactions is important as targeting molecules for therapeutic intervention in one pathway may influence the function of another. Our merged model allows us to predict these apparent “off-target” effects and should permit the design of proper alternative strategies that selectively modulates only the desired pathway. As these signaling pathways are important for T-cell differentiation, our ability to modulate them may enable cellular reprogramming to shift the balance towards a regulatory phenotype for treating autoimmune disease or towards an activated phenotype for enhancing anti-tumor responses.

The recent study by Naldi et al. applies a logical framework to analyze T-cell differentiation [55]. Given the complexity of the system, the authors utilize a model reduction approach to explore T-cell differentiation *in silico*. Since differentiation results from the input of multiple signaling pathways, there is also a great potential for cross-talk. Therefore, it would be interesting to combine individual signaling networks with the differentiation model to see if the combined approach provides a better insight into T-cell differentiation. This would be particularly relevant for predicting the influence of TCR-induced STAT activation upon the signaling networks of the various cytokine receptors. One task would be the conversion of logical models into dynamic ones, which can be performed using the tool developed by [56]. However, one challenge will be to constrain the parameters. In this case, studies on the effects of IL-2 on T-cell proliferation, survival, and population dynamics should be taken into account [8]–[10], [57], [58]. We believe that only by utilizing multiple models with varying levels of complexity can we hope to improve our understanding of T cell biology.

## Supporting Information

Figure S1 **A feasible negative feedback loop.** Usually the cycle of FYN, PAG, CSK, and LCKP1 would lead to an infeasible solution as the activating interactions (solid lines) between the four proteins demand that they are in the same state. This is in conflict with the inhibition of LCKP1 by CSK (dotted red line) which requires that one of them is active and the other inactive. As a consequence one or more of the interactions needs to be classified as a late implication formula. However, the additional input by TCRB allows a feasible solution by creating flexibility in the state of PAG.
(TIF)

Figure S2 **Determination of CD25 and Annexin V/PI by FACS staining.** After resting for 24 hr and before restimulation with IL-2, T-cell blasts were stained with a FITC-coupled anti-CD25 antibody to determine the percentage of CD25-positive cells (A) or a FITC-coupled Annexin V antibody and propidium iodide to determine the number of viable cells (B).
(TIF)

Figure S3 **Dose response of IL-2.** Human T-cell blasts were stimulated by the indicated dilutions of IL-2 [10,000 U]. Cell lysates were analyzed by Western blotting for phosphorylated ERK and STAT3. β-actin was analyzed as loading control.
(TIF)

Figure S4 **The reversible SFK inhibitor PP2 does not fully block AKT.** The blot is a longer exposure of the blots of Figure 3B and 4B demonstrating that the irreversible PI3K inhibitor WM is more efficient than PP2 in blocking SFK-dependent AKT-phosphorylation following IL-2 stimulation.
(TIF)

Figure S5 **The TCR/CD4/CD28 signaling network.** To a large extent the signaling network was published previously in a different graphical layout [3]. The top layer represents input nodes. The bottom layer represents the output, i.e. molecules including transcription factors that become activated. Solid black arrows indicate activating interactions with a black circle denoting AND-connections. For clarity, activating influences with arrows pointing from the bottom to the top are drawn with dashed black lines. Red lines mark inhibitory influences that are expressed as NOT-conditions in the logical network. Note that some connections are based on below-quality-standard data (“potential connections”), but are not highlighted separately (Table S2). Detailed descriptions for the interpretation of each node can be found in Table S1.
(TIF)

Figure S6 **The merged network of TCR/CD4/CD28 and IL-2R signaling.** The top layer represents input nodes. The bottom layer represents the output, i.e. molecules including transcription factors that become activated. Solid black arrows indicate activating interactions with a black circle denoting AND-connections. For clarity, activating influences with arrows pointing from the bottom to the top are drawn with dashed black lines. Red lines mark inhibitory influences that are expressed as NOT-conditions in the logical network. Note that some connections are based on below-quality-standard data (“potential connections”), but are not highlighted separately (Table S2). The nodes specific to the IL-2R and TCR network are shown in blue and green, respectively. Common nodes are highlighted in red; those marked with a thick line are potential mediators of negative cross-regulation identified by the merging process. Newly identified common signaling elements retain their original color, i.e. blue green nodes respectively, but are now marked with a thick line. The new interactions investigated in the study are indicated by bold arrows. A detailed description of each node can be found in Table S1.
(TIF)

Figure S7 **STAT activation after TCR stimulation of mouse T cells.** Primary mouse T cells and mouse T-cell blasts were stimulated as indicated in Protocol S1 and analyzed by Western blotting for the activation of STAT3 and STAT5. Irrelevant lanes have been cut out from the blots.
(TIF)

Figure S8 **IL-2R signaling does not affect TCR expression.** Human T-cell blasts were either stimulated with IL-2 for 30 min or left untreated. The level of TCR (CD3) and IL-2Rα (CD25) surface expression were measured by flow cytometry. Unstained cells are indicated with the broken line, untreated cells are represented with the grey line, and stimulated cells indicated by the black line.
(TIF)

Protocol S1 **Stimulation of mouse T cells.** Method describing the isolation, culture, and stimulation of primary mouse T cells and mouse T-cell blasts.
(PDF)

Text S1 **Model merging as an IFFSAT problem.** Restating the problem of merging two networks as a projection of a larger network, including a formal proof of the algorithmic complexity.
(PDF)

Text S2 **Protocol for Boolean model merging.** A step-by-step protocol for the semi-automated merging of Boolean networks.
(PDF)

Table S1 **List of network components.** The components of the merged TCR-IL-2R network including biological names and interpretation of the ON state.
(PDF)

Table S2 **List of clauses for the merged network.** The implication formulas for each component of the merged network are provided along with references supporting the interactions.
(PDF)

Table S3 **Activation states of the merged network upon different stimuli.** Lists the state vectors for the merged network with TCR and IL-2R stimulation alone or in combination.
(PDF)
