# Supplementary figures and images for: Integrating Signals from the T-Cell Receptor and the Interleukin-2 Receptor
Source: PLoS Comput Biol. 2011 Aug 4;7(8):e1002121. doi: 10.1371/journal.pcbi.1002121 (PMC3150289; doi:10.1371/journal.pcbi.1002121)

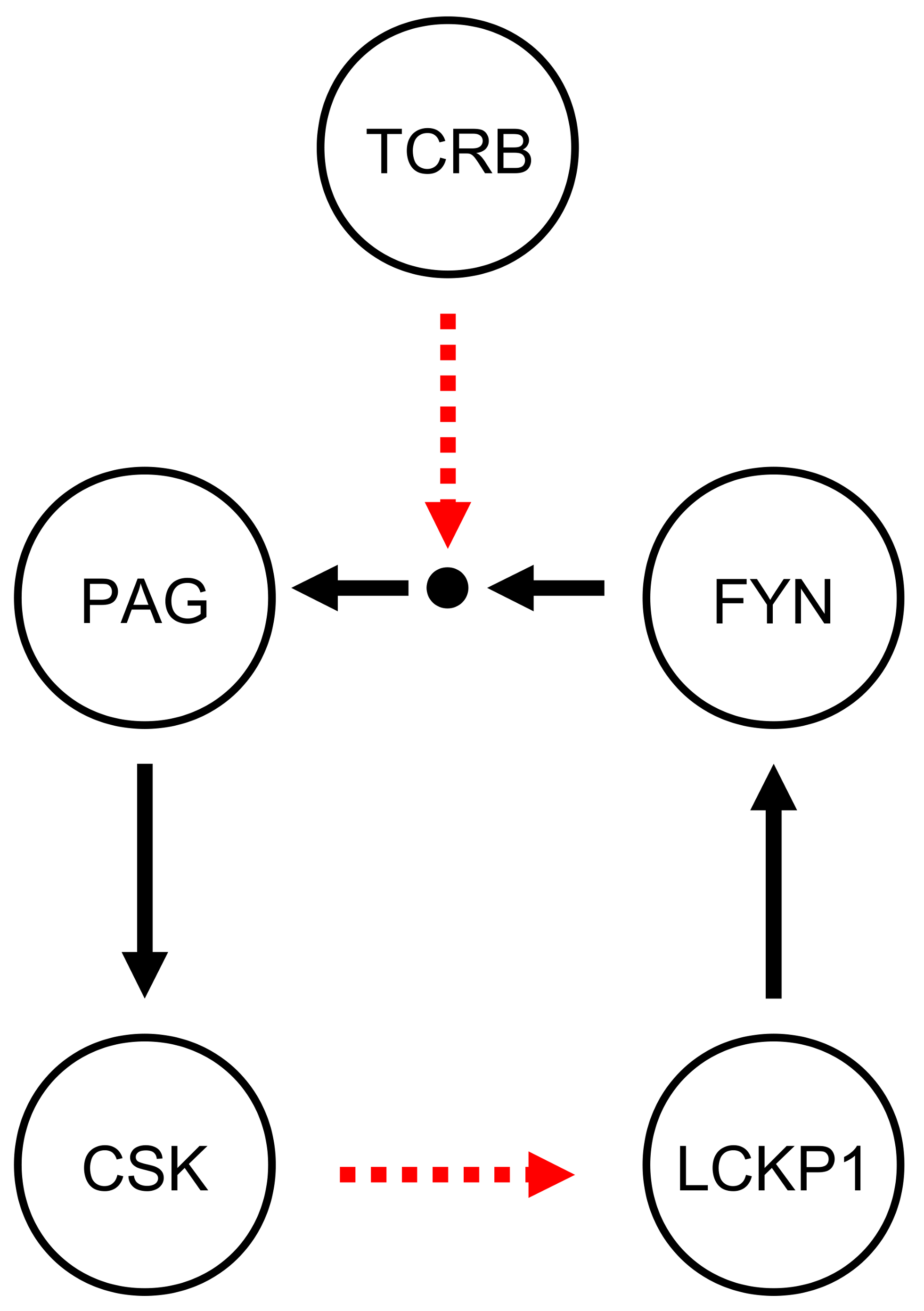

Supplement: Figure S1 — A feasible negative feedback loop. Usually the cycle of FYN, PAG, CSK, and LCKP1 would lead to an infeasible solution as the activating interactions (solid lines) between the four proteins demand that they are in the same state. This is in conflict with the inhibition of LCKP1 by CSK (dotted red line) which requires that one of them is active and the other inactive. As a consequence one or more of the interactions needs to be classified as a late implication formula. However, the additional input by TCRB allows a feasible solution by creating flexibility in the state of PAG. (TIF) [file pcbi.1002121.s001.tif]

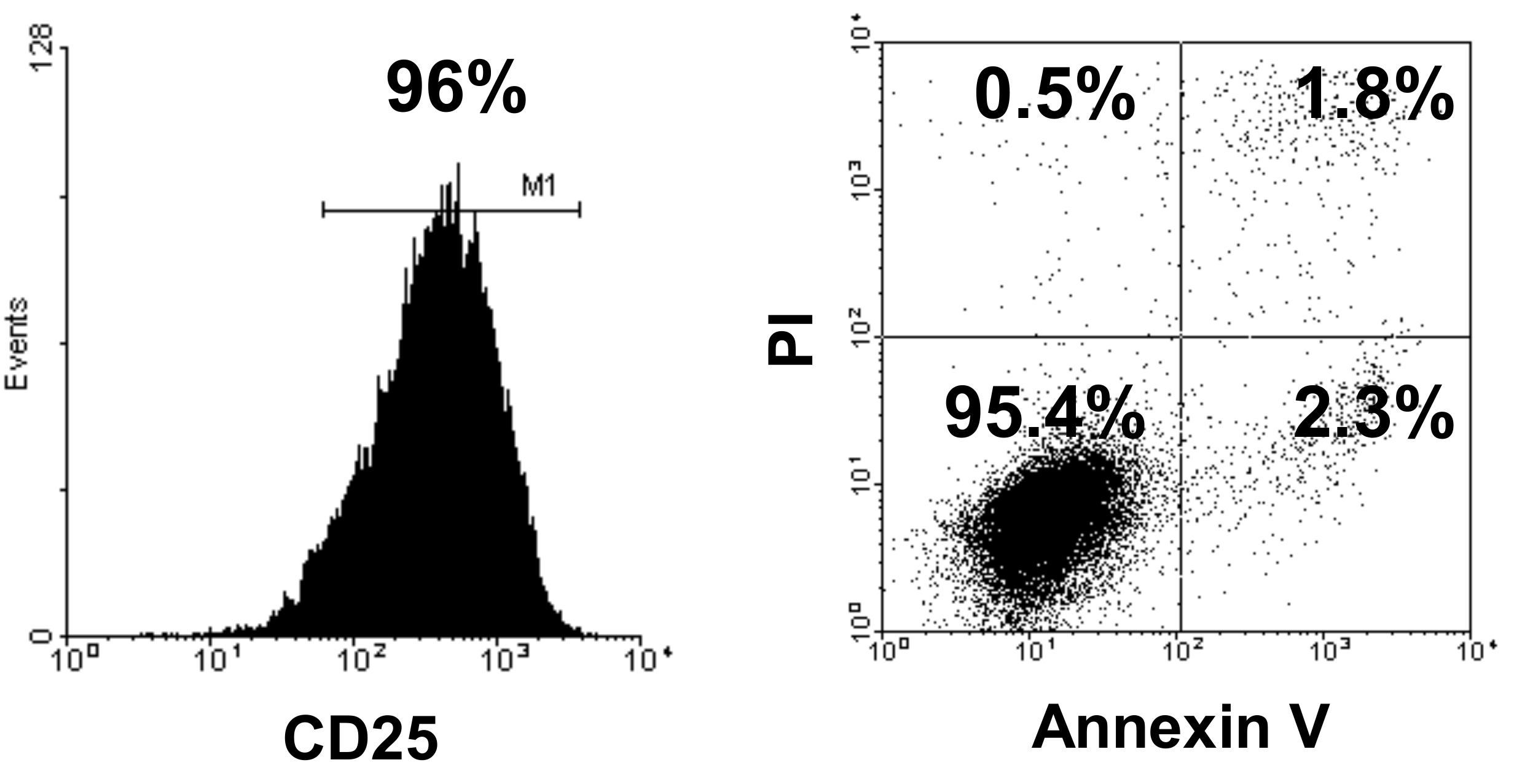

Supplement: Figure S2 — Determination of CD25 and Annexin V/PI by FACS staining. After resting for 24 hr and before restimulation with IL-2, T-cell blasts were stained with a FITC-coupled anti-CD25 antibody to determine the percentage of CD25-positive cells (A) or a FITC-coupled Annexin V antibody and propidium iodide to determine the number of viable cells (B). (TIF) [file pcbi.1002121.s002.tif]

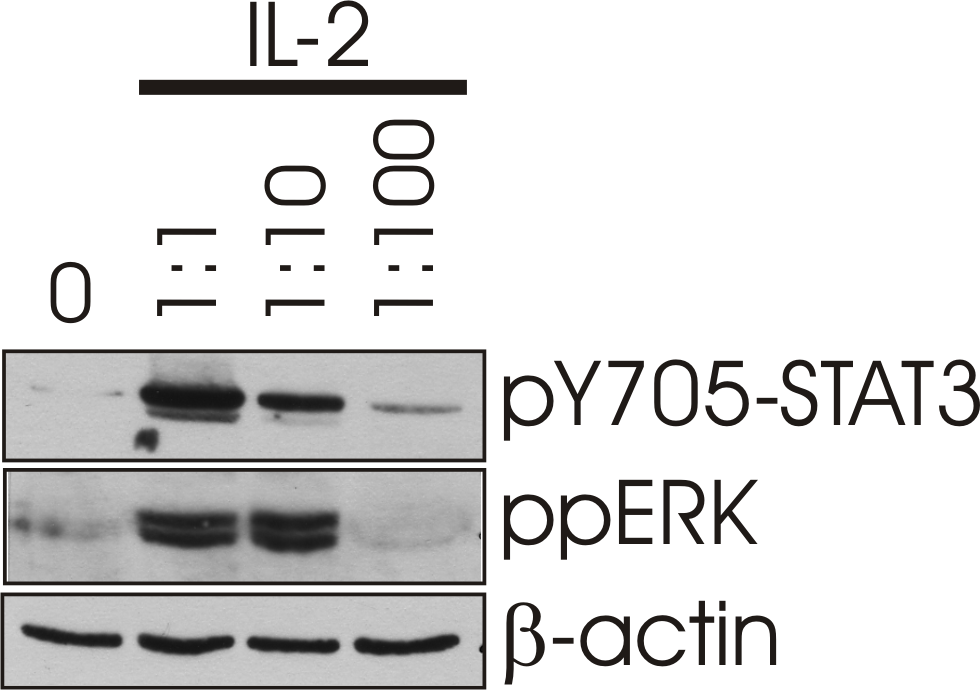

Supplement: Figure S3 — Dose response of IL-2. Human T-cell blasts were stimulated by the indicated dilutions of IL-2 [10,000 U]. Cell lysates were analyzed by Western blotting for phosphorylated ERK and STAT3. β-actin was analyzed as loading control. (TIF) [file pcbi.1002121.s003.tif]

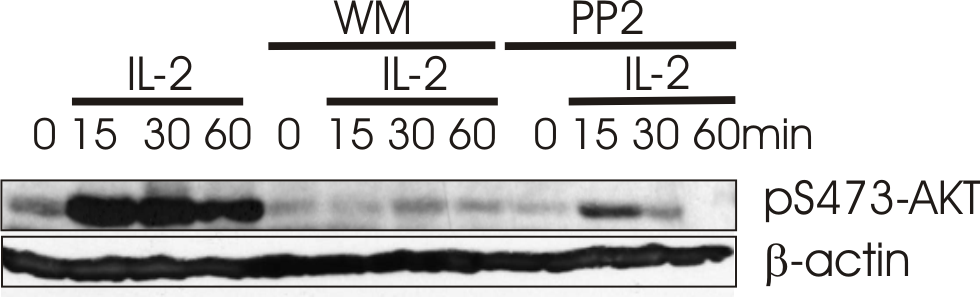

Supplement: Figure S4 — The reversible SFK inhibitor PP2 does not fully block AKT. The blot is a longer exposure of the blots of Figure 3B and 4B demonstrating that the irreversible PI3K inhibitor WM is more efficient than PP2 in blocking SFK-dependent AKT-phosphorylation following IL-2 stimulation. (TIF) [file pcbi.1002121.s004.tif]

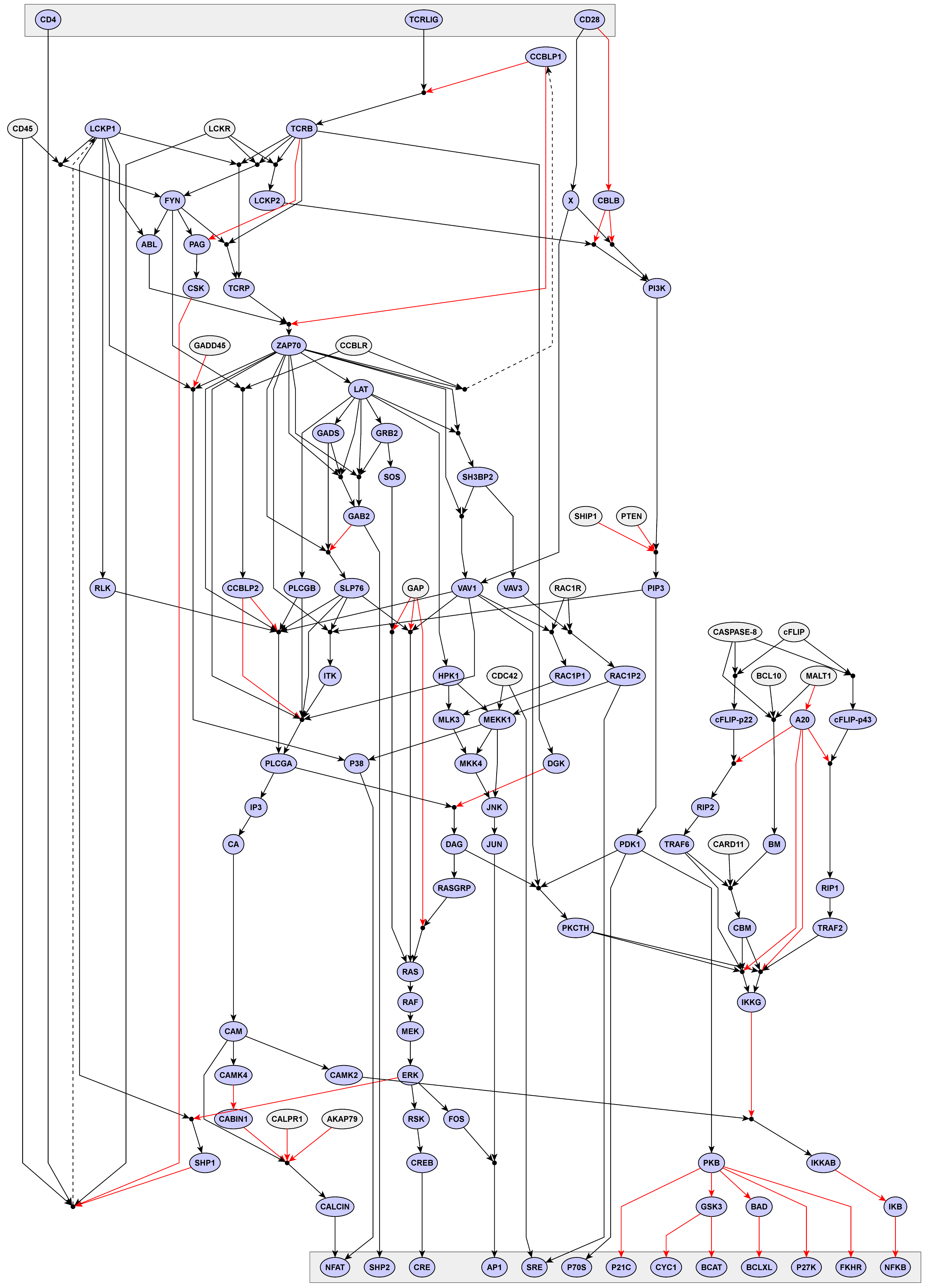

Supplement: Figure S5 — The TCR/CD4/CD28 signaling network. To a large extent the signaling network was published previously in a different graphical layout [3]. The top layer represents input nodes. The bottom layer represents the output, i.e. molecules including transcription factors that become activated. Solid black arrows indicate activating interactions with a black circle denoting AND-connections. For clarity, activating influences with arrows pointing from the bottom to the top are drawn with dashed black lines. Red lines mark inhibitory influences that are expressed as NOT-conditions in the logical network. Note that some connections are based on below-quality-standard data (“potential connections”), but are not highlighted separately (Table S2). Detailed descriptions for the interpretation of each node can be found in Table S1. (TIF) [file pcbi.1002121.s005.tif]

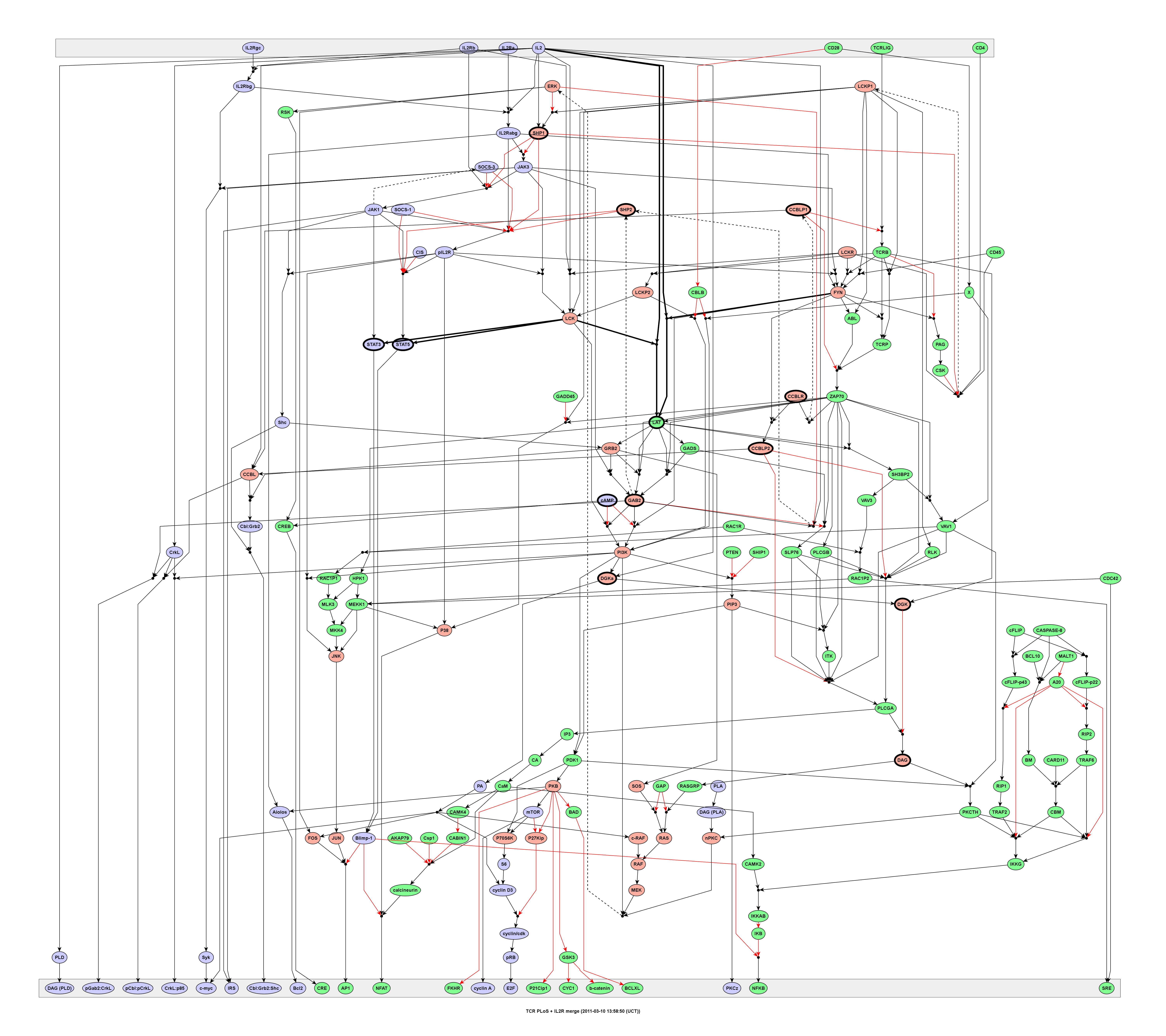

Supplement: Figure S6 — The merged network of TCR/CD4/CD28 and IL-2R signaling. The top layer represents input nodes. The bottom layer represents the output, i.e. molecules including transcription factors that become activated. Solid black arrows indicate activating interactions with a black circle denoting AND-connections. For clarity, activating influences with arrows pointing from the bottom to the top are drawn with dashed black lines. Red lines mark inhibitory influences that are expressed as NOT-conditions in the logical network. Note that some connections are based on below-quality-standard data (“potential connections”), but are not highlighted separately (Table S2). The nodes specific to the IL-2R and TCR network are shown in blue and green, respectively. Common nodes are highlighted in red; those marked with a thick line are potential mediators of negative cross-regulation identified by the merging process. Newly identified common signaling elements retain their original color, i.e. blue green nodes respectively, but are now marked with a thick line. The new interactions investigated in the study are indicated by bold arrows. A detailed description of each node can be found in Table S1. (TIF) [file pcbi.1002121.s006.tif]

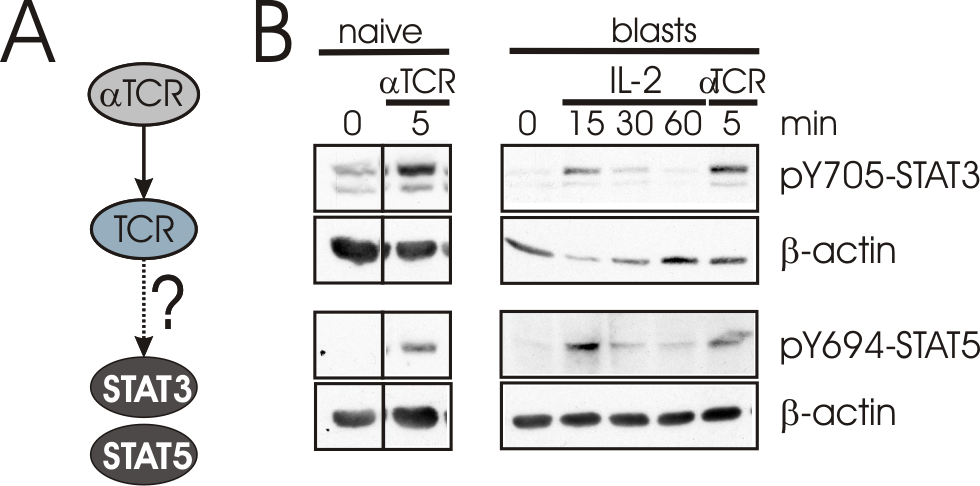

Supplement: Figure S7 — STAT activation after TCR stimulation of mouse T cells. Primary mouse T cells and mouse T-cell blasts were stimulated as indicated in Protocol S1 and analyzed by Western blotting for the activation of STAT3 and STAT5. Irrelevant lanes have been cut out from the blots. (TIF) [file pcbi.1002121.s007.tif]

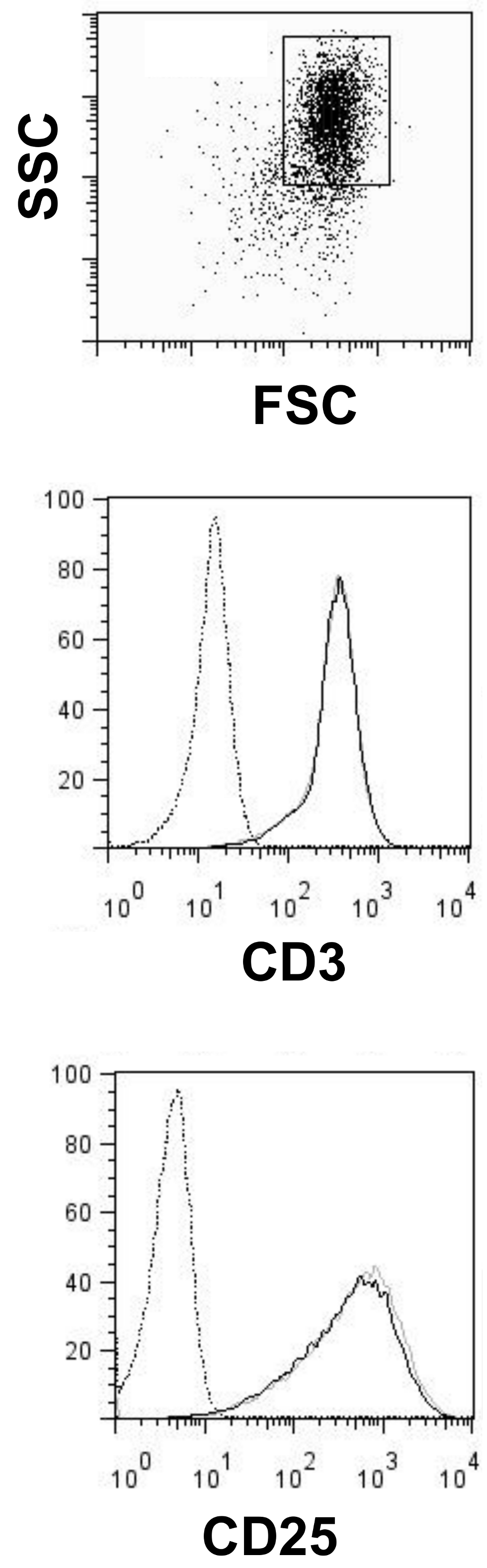

Supplement: Figure S8 — IL-2R signaling does not affect TCR expression. Human T-cell blasts were either stimulated with IL-2 for 30 min or left untreated. The level of TCR (CD3) and IL-2Rα (CD25) surface expression were measured by flow cytometry. Unstained cells are indicated with the broken line, untreated cells are represented with the grey line, and stimulated cells indicated by the black line. (TIF) [file pcbi.1002121.s008.tif]
