# Supplementary material for: Integrating Signals from the T-Cell Receptor and the Interleukin-2 Receptor
Source: PLoS Comput Biol. 2011 Aug 4;7(8):e1002121. doi: 10.1371/journal.pcbi.1002121 (PMC3150289; doi:10.1371/journal.pcbi.1002121)
Supplement: Protocol S1 — Stimulation of mouse T cells. Method describing the isolation, culture, and stimulation of primary mouse T cells and mouse T-cell blasts. (PDF) [file pcbi.1002121.s009.pdf]

Protocol S1

C57/Bl.6 mice of age approximately 8 weeks were sacrificed, spleens were taken out and mashed through a 70 µm cell strainer, on ice. Cells were resuspended thoroughly, washed twice in ice-cold PBS (without Ca^{2+} and Mg^{2+}) and T cells were isolated by negative selection with the Pan T Cell Isolation MACS kit for mouse cells (Miltenyi, Cat # 130-090-861), according to the manufacturer's instructions. After washing in PBS, T cells were plated at a density of 0,5 x 10^6 cells per well in a 24-well plate (precoated for 4 h, 37°C or overnight, 4°C with 5 µg/ml anti-mouse CD28 antibody (BD Pharmingen, Cat # 553295) and 3 µg/ml anti-mouse CD3 antibody (BD Pharmingen, Cat # 553239)), in RPMI supplemented with penicillin and streptavidin, 10% FCS and 50 µM 2-mercaptoethanol, as wells as 10 ng/ml IL-2 (Peprotech, Cat # 200-02). Cells were thus cultivated in a humidified incubator at 37°C for 48h, then washed in PBS, transferred to a new plate in the same medium without IL-2 and rested at 37°C for 24h. For the stimulations, cells were washed from the medium with PBS, and stimulations were performed in RPMI with IL-2 (10 ng/ml) at 37°C for the indicated time points. Reactions were stopped with ice-cold TBS and cells were lysed as described for human T cells.
