# Supplementary material for: Integrating Signals from the T-Cell Receptor and the Interleukin-2 Receptor
Source: PLoS Comput Biol. 2011 Aug 4;7(8):e1002121. doi: 10.1371/journal.pcbi.1002121 (PMC3150289; doi:10.1371/journal.pcbi.1002121)
Supplement: Text S1 — Model merging as an IFFSAT problem. Restating the problem of merging two networks as a projection of a larger network, including a formal proof of the algorithmic complexity. (PDF) [file pcbi.1002121.s010.pdf]

Text S1.

Model Merging

Let us formalize the question as follows: Given a network N with component set C and statement set S, and let a subset of components K of C be given. We ask "Is there a network N' on the set of components K with statement set S' so that every feasible solution in N' can be extended to a feasible solution in N?" (Q1). To verify a solution to (Q1) one needs to check whether for a given network N' these requirements are fulfilled, but this problem is in general provably hard (Π_2^P -complete) [1]. Furthermore, the computation of N' is difficult as it requires the computation of the logical projection of a system onto the set of components K, which may be exponential in the number of components [2]. It may be the case that the projection no longer has the form we required for a logical signaling network, namely that all statements are in IFF-form. In fact, it could be that no formulation with IFF-clauses on the set of statements K is possible. If we consider the artificial network that consists only of the IFF-clause *cAMP OR RSK ↔CREB*, a projection onto cAMP and CREB leads to the implication *cAMP → CREB*. But assuming the slightly bigger network that contains the formulas *cAMP OR RSK ↔ CREB, ERK ↔ RSK*, the projection of RSK leads to the IFF-clause *cAMP OR ERK ↔ CREB.* For computational purposes this is not of importance, but for purposes of visualization and examination by biological experts arbitrary logical formulas are unsuitable. We therefore relax the question to the following: "What is a minimal set of components K' which contains K, so that a set of IFF-statements S' exists such that every feasible solution of this network can be extended to a solution of N in this network?" (Q2) Clearly, K' can be chosen equal to C, so we know a trivial upper boundary. Hence, if we can check whether a projection can be written in IFF-form, we can solve (Q2) as a monotone generation problem [3]. A survey of these can be found in [4].

Complexity of Signaling Network Projection

Definition: (polynomial hierarchy)

The polynomial hierarchy is defined as

$$\Sigma_0^P := \Pi_0^P := P \; ; \; \Sigma_{k+1}^P := NP^{\Sigma_k^P} \; ; \; \Pi_{k+1}^P := co-\Sigma_{k+1}^P \, ,$$

where Σ_{k+1}^P is the class of all problems which can be decided non-deterministically in polynomial time with the help of an oracle for a problem in Σ_k^P , i.e. given a certificate for the positive answer to an instance of a problem in Σ_{k+1}^P , it can be verified in polynomial time using an oracle for a

problem in Σ_k^P . The class Π_{k+1}^P contains all decision problems whose complements are in Σ_{k+1}^P . For instance, $\Sigma_1^P = NP$ and $\Pi_1^P = co-NP$. See [5] for more details.

Proposition: The verification of a given projection N′ is Π_2^P -complete.

Proof: Let N be an IFFSAT instance representing a signaling network and K its components. To verify that a given N' is a projection of N onto a subset of components $K' \subseteq K$ the answer to the following decision problem must be YES:

$$\forall x \in \{0,1\}^{|K'|} \left(N'(x) \leftrightarrow \exists y \in \{0,1\}^{|K \setminus K'|} N(x,y) \right) \quad (1)$$

where we denote by $F(x)$ the truth value of a formula F for a given truth assignment x . To show that (1) is in Π_2^P , we have to argue that the check whether a given polynomial size certificate is a counterexample can be done in polynomial time using an NP oracle. To see this, we take x as the certificate. Whenever $N'(x)$ evaluates to FALSE, it is to check whether there exists y such that $N(x,y)$ is TRUE, which is the classical SAT problem and therefore NP -complete. Thus, given a SAT oracle, the counterexample is verified in polynomial time. If $N'(x)$ evaluates to TRUE, it is to check whether for all y $N(x,y)$evaluates to FALSE, which is equivalent to a NO answer to SAT. Hence, given a SAT oracle, we can test the counterexample in polynomial time and therefore (1) is in Π_2^P .

It remains to argue that (1) is also complete in Π_2^P . We transform the following prototypical Π_2^P -complete problem to our instance.

$$\forall x \in \{0,1\}^k \, \exists y \in \{0,1\}^n \, F(x,y) \quad (2)$$

where $F(x,y)$ is in IFFSAT form. (IFFSAT form can be assumed since IFFSAT was shown to be NP -complete by reduction from 3-SAT [6]). Now we choose F to be N and N' to be $\{x_i \leftrightarrow x_i\}$ for some component x_i of x . This is a tautology and thus $N'(x)$ is always TRUE. Thus, we can formulate (2) equivalently as

$$\forall x \in \{0,1\}^k \left(N'(x) \leftrightarrow \exists y \in \{0,1\}^n N(x,y) \right)$$

which completes the proof.

1. Truemper K (2004) Design of Logic-based Intelligent Systems. John Wiley & Sons.
2. Hooker JN (1992) Logical Inference and Polyhedral Projection. In: Proceeedings, Computer Science Logic Workshop (CSL’91), Lecture Notes in Computer Science 626: 184-200.
3. Fredman ML, Khachiyan L (1996) On the complexity of dualization of monotone disjunctive normal forms. J. Algorithms 21: 618–628. doi:http://dx.doi.org/10.1006/jagm.1996.0062
4. Eiter T, Makino K, Gottlob G (2008) Computational aspects of monotone dualization: A brief survey. Discrete Appl. Math. 156: 2035–2049. doi:http://dx.doi.org/10.1016/j.dam.2007.04.017
5. Büning HK, Lettmann T (1999) Propositional Logic: Deduction and Algorithms. Cambridge University Press.
6. Haus U-U, Truemper K, Weismantel R (2008) Linear Satisfiability Algorithm for 3CNF Formulas of Certain Signaling Networks. JSAT 6: 13-32.
