# Supplementary material for: Integrating Signals from the T-Cell Receptor and the Interleukin-2 Receptor
Source: PLoS Comput Biol. 2011 Aug 4;7(8):e1002121. doi: 10.1371/journal.pcbi.1002121 (PMC3150289; doi:10.1371/journal.pcbi.1002121)
Supplement: Text S2 — Protocol for Boolean model merging. A step-by-step protocol for the semi-automated merging of Boolean networks. (PDF) [file pcbi.1002121.s011.pdf]

Text S2.

Protocol for Boolean model merging.

Steps marked with a `*' are not fully automatic and require intervention from the user.

1. Identify those components that are not present in both parent networks.
2. Check whether those components have alternative names (using common databases like UNIPROT or STRING)
3. (*) Check whether components with matching alternative names actually denote the same thing in both parent networks, and whether the decisions about their ON/OFF-interpretation and discretization agree. If so, decide on a common name and rename them in both parents.

3a It is possible that components of one parent network are a subset of components in the other parent, i.e. one component is actually representing a whole class of molecules as it is frequently the case for isoforms of proteins. If this applies, add a statement to ensure that activation of the specific component activates the more general class, such that for example an isoform is representative for the activation of the whole class.

4. (*) Decide if all components that occur in only one parent should be propagated to the child. If not, delete them.
5. (*) Check that all components that occur in both parents actually designate the same object. If not, rename uniquely. For example the IL-2R network contained the production of DAG species via a PLD-dependent pathway. The TCR network also produces DAG but via the PLCγ1-pathway. Therefore the source for DAG is different and may indeed result in different DAG forms and consequently in different sets of downstream molecules activated by those DAG species [13].
6. Copy all components to the child.
7. Copy all statements that are identical in both parents to the child.
8. (*) For each remaining statement check whether it can be merged. This may entail checking the literature again, or performing additional experiments. If, for example, in one parent we have the statement *A* → *C*, and in the other *A AND B* → *C*, then one needs to check whether the dependency of C on B is always true, or holds only under some extra condition. This extra condition may be stimulation of a receptor that is only present in this parent. In the first case the stronger statement can be copied to the child, in the other case we label each statement with a precondition specific to the particular parent, e.g. *IL2 AND GAB2 AND NOT ERK* → *SHP2* as the negative feedback from *ERK* on *GAB2* seems to be specific for

IL-2 signaling. For the TCR network the clause reads only *GAB2* → *SHP2*.

9. Copy all remaining statements.

After the merging procedure both parent networks are projections of the child network in the sense of (Q1) in the main text.
