# Supplementary material for: Integrating Signals from the T-Cell Receptor and the Interleukin-2 Receptor
Source: PLoS Comput Biol. 2011 Aug 4;7(8):e1002121. doi: 10.1371/journal.pcbi.1002121 (PMC3150289; doi:10.1371/journal.pcbi.1002121)
Supplement: Table S1 — List of network components. The components of the merged TCR-IL-2R network including biological names and interpretation of the ON state. (PDF) [file pcbi.1002121.s012.pdf]

Table S1

The ON-interpretation is as follows:

complex	formation of the complex, recruitment
kinases	kinase activity, including proper localization
phosphatases	phosphatase activity, including proper localization
adaptor	recruitment
transcription factor	nuclear localization and transcriptional activity
secondary messenger	increased presence of the molecule
ligand	presence
E3 uqbiquitin ligase	ubiquitin transfer
GEF	nucleotide exchange activity
GAP	binding to small GTPase and stimulation of GTPase activity
phospholipase	lipid hydrolysis

Name	Full Name	Type or different ON-Interpretation
A20	zinc finger A20 domain-containing protein 1/ cellular zinc finger anti-NF-kappa-B protein	de-ubiquitinating enzyme
ABL	Abelson protein tyrosine kinase	kinase
Aiolos	zinc finger protein Aiolos	transciption factor
AKAP79	adaptorkinase (PRKA) protein anchor 79	adaptor
AP1	v-jun sarcoma virus 17 oncogene homology	transciption factor
b-catenin	catenin (cadherin-associated protein) beta 1	
BAD	BCL2-antagonist of cell death	unphosphorylated active form
BCL10	B-cell CLL/lymphoma 10	expression
Bcl2	B-cell CLL/lymphoma 2	expression
BCLXL	BCL2-like 1	expression
Blimp-1	beta-interferon gene positive- regulatory domain I binding factor	transciption factor
BM	complex of CARMA1 and BCL10	complex
c-myc	v-myc myelocytomatosis viral oncogene homolog (avian)	transciption factor
c-RAF	v-raf-1 murine leukemia viral oncogene homolog 1	kinase
CA	calcium	secondary messenger
CABIN1	calcineurin-binding protein	adaptor
calcineurin	calcineurin	phosphatase
CaM	calmodulin	Ca^{2+}-bound active form
CAMK2	calcium/calmodulin-dependent protein kinase (CaM kinase) II	kinase

CAMK4	calcium/calmodulin-dependent protein kinase IV	kinase
cAMP	cyclic AMP	secondary messenger
CARD11	caspase recruitment domain family, member 11	recruitment
CASPASE-8	caspase 8	proteolytic activity
Cbl:Grb2	complex of Cbl and Grb2	complex
Cbl:Grb2:Shc	complex of Cbl, Grb2, and Shc	complex
CBLB	Cas-Br-M (murine) ecotropic retroviral transforming sequence b	E3 ubiquitin ligase
CBM	complex of CARD11, CARMA1, and BCL10	complex
CCBL	Casitase B-lineage lymphoma protooncogene (all pools)	E3 ubiquitin ligase
CCBLP1	Casitase B-lineage lymphoma protooncogene (ZAP70-dependent pool)	E3 ubiquitin ligase
CCBLP2	Casitase B-lineage lymphoma protooncogene (ZAP70-dependent pool)	E3 ubiquitin ligase
CCBLR	Casitase B-lineage lymphoma protooncogene (inactive reseroir)	E3 ubiquitin ligase
CD28	CD28 antigen (Tp44)	receptor
CD4	CD4 antigen (p55)	receptor
CD45	protein tyrosine phosphatase, receptor type, C	phosphatase
CDC42	cell division cycle 42 (GTP binding protein, 25kDa)	small GTPase
cFLIP	caspase-related inducer of apoptosis	presence
cFLIP-p22	cFLIP, short proteolytic fragment	presence
cFLIP-p43	cFLIP, short proteolytic fragment	presence
CIS	cytokine-inducible SH2-containing	binding to JAKs
CRE	cAMP responsive element binding protein 1	nuclear localization
CREB	cAMP responsive element	transcitpion
CrkL	Crk-like protein	adaptor
CrkL:p85	complex of p85 (PI3K subunit) and CrkL	complex
CSK	c-src tyrosine kinase	kinase
Csp1	Calcipressin 1	binding to calcineurin
CYC1	cytochrome c-1	release from mitochondria
cyclin A	cyclin A	expression
cyclin D3	cyclin D3	expression
cyclin/cdk	complex of cyclin and cylin-dependent kinase	kinase
DAG	diacylglycerol	secondary messenger
DAG (PLA)	diacylglycerol (PLA-derived species)	secondary messenger
DAG (PLD)	diacylglycerol (PLD-pathway-derived species)	secondary messenger
DGK	diacylglycerol kinase (all isoforms)	kinase
DGKa	diacylglycerol kinase alpha	kinase

E2F	PRB-binding protein E2F	transciption factor
ERK	Extracellular signal-regulated kinases, mitogen-activated protein kinase 1	kinase
FKHR	forkhead box O1A	transciption factor
FOS	FOS-like antigen	expression
FYN	FYN oncogene related to SRC, FGR, YES	kinase
GAB2	GRB2-associated binding protein 2	adaptor
GADD45	growth arrest and DNA-damage-inducible, alpha	presence
GADS	GRB2-related adaptor protein 2	adaptor
GAP	GTPase-activating protein	Ras-GAP
GRB2	growth factor receptor-bound protein 2	adaptor
GSK3	glycogen synthase kinase 3	kinase
HPK1	Hematopoietic progenitor kinase 1, mitogen-activated protein kinase kinase kinase kinase 1	kinase
IKB	inhibitor protein IkappaB	binding to NFkappaB
IKKAB	inhibitor of kappa light polypeptide gene enhancer in B-cells, kinase alpha and kinase beta	kinase
IKKG	inhibitor of kappa light polypeptide gene enhancer in B-cells, kinase gamma	kinase
IL2	interleukin-2	ligand
IL2Ra	IL-2 receptor alpha chain	receptor
IL2Rabg	IL-2 receptor complex: alpha, beta, and common gamma chain	receptor
IL2Rb	IL-2 receptor beta chain	receptor
IL2Rbg	medium affinity IL-2 receptor: beta and common gamma chain	receptor
IL2Rgc	IL-2 receptor common gamma chain	receptor
IP3	inositol 1,4,5-trisphosphate	secondary messenger
IRS	insulin receptor susbtrate	adaptor
ITK	IL2-inducible T-cell kinase	kinase
JAK1	janus kinase 1	kinase
JAK3	janus kinase 2	kinase
JNK	mitogen-activated protein kinase 8	kinase
JUN	v-jun sarcoma virus 17 oncogene homolog	expression
LAT	linker for activation of T cells	adaptor
LCK	lymphocyte-specific protein tyrosine kinase (all pools)	kinase
LCKP1	lymphocyte-specific protein tyrosine kinase (CD4-dependent pool)	kinase
LCKP2	lymphocyte-specific protein tyrosine kinase (CD28/TCR-dependent pool)	kinase

LCKR	lymphocyte-specific protein tyrosine kinase (inactive reservoir)	kinase
MALT1	mucosa associated lymphoid tissue lymphoma translocation gene 1	presence
MEK	mitogen-activated protein kinase kinase	kinase
MEKK1	mitogen-activated protein kinase kinase kinase	kinase
MKK4	mitogen-activated protein kinase kinase 4	kinase
MLK3	mitogen-activated protein kinase kinase kinase 11	kinase
mTOR	mammalian target of rapamycin	kinase
NFAT	nuclear factor of activated T-cells, cytoplasmic, calcineurin-dependent 1	transciption factor
NFKB	nuclear factor of kappa light polypeptide gene enhancer in B-cells 1, 2	transciption factor
nPKC	protein kinase C, novel isoforms	kinase
P21Cip1	cyclin-dependent kinase inhibitor 1A (p21, Cip1)	binding to cdk
P27Kip	cyclin-dependent kinase inhibitor 1B (p27,Kip1)	binding to cdk
P38	mitogen-activated protein kinase 14	kinase
P70S6K	ribosomal protein S6 kinase, 70kDa,polypeptide 1	kinase
PA	phosphatidic acid	secondary messenger
PAG	phosphoprotein associated with glycosphingolipid microdomains 1	adaptor
pCbl:pCrkL	phosphotyrosine-depndent complex of Cbl and CrkL	complex
PDK1	3-phosphoinositide dependent protein kinase-1	kinase
pGab2:CrkL	phosphotyrosine-dependent complex of Gab2 and CrkL	complex
PI3K	phosphoinositide-3-kinase, catalytic, alpha polypeptide	kinase
pIL2R	IL-2 recepotr complex, phosphorlated at beta chain	receptor
PIP3	phosphatidylinositol (3,4,5)-trisphosphate	secondary messenger
PKB	v-akt murine thymoma viral oncogene homolog 1	kinase
PKCTH	protein kinase C theta	kinase
PKCz	protein kinase C zeta	kinase
PLA	phospholipaseadaptor	phospholipase
PLCGA	phospholipase C gamma 1, active form	phospholipase

PLCGB	phospholipase C gamma 1, recruited to LAT but enzymatically inactive	phospholipase
PLD	phospholipase D	phospholipase
pRB	retinoblastoma tumor suppressor protein	
PTEN	phosphatase and tensin homolog (mutated in multiple advanced cancers 1)	phosphatase
RAC1P1	ras-related C3 botulinum toxin substrate 1 (rho family, small GTP binding protein Rac1) (Vav-1 dependent pool)	small GTPase
RAC1P2	ras-related C3 botulinum toxin substrate 1 (rho family, small GTP binding protein Rac1) (Vav-3 dependent pool)	small GTPase
RAC1R	ras-related C3 botulinum toxin substrate 1 (rho family, small GTP binding protein Rac1) (inactive reservoir)	small GTPase
RAF	v-raf-1 murine leukemia viral oncogene (all isoforms)	kinase
RAS	small GTPase Ras	small GTPase
RASGRP	Ras guanyl releasing protein 1	Ras-GEF
RIP1	receptor interacting protein kinase 1	kinase
RIP2	receptor interacting protein kinase 1	kinase
RLK	TXK tyrosine kinase	kinase
RSK	ribosomal protein S6 kinase, 90kDa, polypeptide 1	kinase
S6	ribosomal protein S6	regulation of translation
SH3BP2	SH3-domain binding protein 2	adaptor
Shc	SH2 domain-containing-transforming protein C	adaptor
SHIP1	inositol polyphosphate-5-phosphatase, 145kDa	phosphatase
SHP1	protein tyrosine phosphatase, nonreceptor type 6	phosphatase
SHP2	protein tyrosine phosphatase, nonreceptor type 11 (Noonan syndrome 1)	phosphatase
SLP76	lymphocyte cytosolic protein 2 (SH2 domain containing leukocyte protein of 76kDa)	adaptor
SOCS-1	suppressor of cytokine signaling 1	binding to JAKs
SOCS-3	suppressor of cytokine signaling 3	binding to JAKs
SOS	Son of Sevenless	Ras-GEF
SRE	serum responsive element	transciption factor
STAT3	signal transducer and activator of transcription 3	transciption factor
STAT5	signal transducer and activator of transcription 3	transciption factor

Syk	spleen tyrosine kinase	kinase
TCRB	T-cell Receptor (bound to ligand)	receptor
TCRLIG	ligand of T cell receptor (peptide-MHC complex or antibody)	ligand
TCRP	T-cell Receptor phosphorylated	receptor
TRAF2	tumor necrosis factor type 2 receptor associated protein 3	E3 ubiquitin ligase
TRAF6	tumor necrosis factor type 2 receptor associated protein 6	E3 ubiquitin ligase
VAV1	Vav 1 oncogene	Rac-GEF
VAV3	Vav 3 oncogene	Rac-GEF
X	Non-identified kinase involved in CD28-mediated signaling	kinase
ZAP70	zeta-chain (TCR) associated protein kinase 70kDa	kinase
