# Supplementary material for: Integrating Signals from the T-Cell Receptor and the Interleukin-2 Receptor
Source: PLoS Comput Biol. 2011 Aug 4;7(8):e1002121. doi: 10.1371/journal.pcbi.1002121 (PMC3150289; doi:10.1371/journal.pcbi.1002121)
Supplement: Table S2 — List of clauses for the merged network. The implication formulas for each component of the merged network are provided along with references supporting the interactions. (PDF) [file pcbi.1002121.s013.pdf]

Table S2

To save space we make use of the common mathematical notation ¬ for NOT, ∨ for OR, and ∧ for AND.

#	Statement	*tau*	References
1	(¬ A20) ∧ CBM ∧ PKCTH ∧ TRAF2 → IKKG	1	[1]
2	(¬ A20) ∧ CBM ∧ PKCTH ∧ TRAF6 → IKKG	1	[1, 2, 3, 4, 5, 6, 7, 8, 9, 10, 11]
3	(¬ A20) ∧ cFLIP-p22 → RIP2	1	[12]
4	(¬ A20) ∧ cFLIP-p43 → RIP1	1	[11, 13, 14, 15]
5	ABL ∧ (¬ CCBLP1) ∧ TCRP → ZAP70	1	[16]
6	Aiolos → Bcl2	1	[17, 18, 19]
7	(¬ AKAP79) ∧ (¬ CABIN1) ∧ CaM ∧ (¬ Csp1)→ calcineurin	1	[20, 21, 22]
8	(¬ BAD) → BCLXL	1	[23, 24]
9	BCL10 ∧ CASPASE-8 ∧ MALT1 → BM	1	[15, 25, 26]
10	(¬ Blimp-1) ∧ calcineurin ∧ P38 → NFAT	1	[27, 28, 29, 30]
11	(¬ Blimp-1) ∧ FOS ∧ JUN → AP1	1	[27, 31]
12	(¬ Blimp-1) ∧ (¬ IKB) → NFKB	1	[27, 31]
13	BM ∧ CARD11 ∧ TRAF6 → CBM	1	[1, 13]
14	c-RAF → RAF	1	isoform relation
15	CA → CaM	1	[32]
16	CaM → CAMK2	1	[33]
17	CaM → CAMK4	1	[34]
18	CAMK2 ∧ IKKG → IKKAB	1	[5, 35, 36]
19	(¬ CAMK4) → CABIN1	1	[37]
20	cAMP → CREB	1	[38]
21	(¬ cAMP) ∧ FYN ∧ GAB2 → PI3K	1	[39, 40]
22	(¬ cAMP) ∧ GAB2 ∧ LCK → PI3K	1	[40, 41, 42, 43, 44, 45, 46, 47, 48, 49]
23	CASPASE-8 ∧ cFLIP → cFLIP-p22	1	[14]
24	CASPASE-8 ∧ cFLIP → cFLIP-p43	1	[14]
25	Cbl:Grb2 ∧ Shc → Cbl:Grb2:Shc	1	[50]
26	(¬ CBLB) ∧ LCKP2 → PI3K	1	[51, 52]
27	(¬ CBLB) ∧ X → PI3K	1	[53, 54, 55, 56, 57, 58]
28	CCBL ∧ CrkL → pCbl:pCrkL	1	[50]
29	CCBL ∧ GRB2 → Cbl:Grb2	1	[50]
30	CCBLP1 ∨ CCBLP2 → CCBL	1	pool relation
31	(¬ CCBLP1) ∧ TCRLIG → TCRB	1	[27]
32	(¬ CCBLP2) ∧ ITK ∧ PLCGB ∧ SLP76 ∧ VAV1 ∧ ZAP70 → PLCGA	1	[59, 60]
33	(¬ CCBLP2) ∧ PLCGB ∧ RLK ∧ SLP76 ∧ VAV1 ∧ ZAP70 → PLCGA	1	[59, 60]
34	CCBLR ∧ FYN → CCBLP2	2	[60]
35	CCBLR ∧ ZAP70 → CCBLP1	2	[61]
36	(¬ CD28) → CBLB	2	[62]

37	CD28 → X	1	[63, 64, 65]
38	CD4 ∧ CD45 ∧ (¬ CSK) ∧ LCKR ∧ (¬ SHP1) → LCKP1	1	[66]
39	CD45 ∧ LCKP1 → FYN	1	[67]
40	CDC42 → MEKK1	1	[68, 69]
41	CDC42 → SRE	1	[70]
42	(¬ CIS) ∧ JAK1 ∧ pIL2R ∧ (¬ SHP2) ∧ (¬ SOCS-1) → STAT5	1	[71, 72, 73, 74, 75, 76, 77, 78]
43	CREB → CRE	1	[38]
44	CrkL ∧ GAB2 → pGab2:CrkL	1	[49, 50]
45	CrkL ∧ PI3K → CrkL:p85	1	[48]
46	cyclin D3 ∧ (¬ P27Kip) → cyclin/cdk	1	[79, 80, 81]
47	cyclin/cdk → pRB	1	[79, 80, 81]
48	DAG → RASGRP	1	[82, 83, 84]
49	DAG ∧ PDK1 ∧ VAV1 → PKCTH	1	[85, 86, 87]
50	DAG (PLA) → nPKC	1	[88, 89, 90, 91, 92, 93, 94]
51	DAG (PLD) → nPKC	1	[88, 89, 90, 91, 92, 93, 94, 95, 96]
52	(¬ DGK) ∧ PLCGA → DAG	1	[27, 97]
53	DGKa → DGK	1	isoform relation
54	DGKa → PA	1	[98, 99, 100]
55	ERK → FOS	1	[27, 101]
56	ERK → RSK	1	[102]
57	(¬ ERK) ∧ GAB2 ∧ IL2 → SHP2	1	[48, 49, 78, 103, 104, 105]
58	(¬ ERK) ∧ LCKP1 → SHP1	2	[106, 107]
59	FYN → ABL	1	[16]
60	FYN ∧ IL2 → LAT	1	own result
61	FYN ∧ (¬ TCRB) → PAG	2	[108, 109]
62	FYN ∧ TCRB → TCRP	1	[110]
63	GAB2 → SHP2	1	[78]
64	(¬ GAB2) ∧ GADS ∧ ZAP70 → SLP76	1	[111, 112, 113, 114]
65	(¬ GADD45) ∧ LCKP1 ∧ ZAP70 → P38	1	[115, 116, 117]
66	GADS ∧ LAT ∧ ZAP70 → GAB2	2	[111, 112]
67	(¬ GAP) ∧ RASGRP → RAS	1	[83, 118]
68	(¬ GAP) ∧ SOS → RAS	1	[83, 101, 118, 119, 120, 121]
69	GRB2 → SOS	1	[121, 122]
70	GRB2 ∧ JAK3 → GAB2	1	[103, 123, 124]
71	GRB2 ∧ LAT ∧ ZAP70 → GAB2	2	[111, 112]
72	(¬ GSK3) → b-catenin	1	[125]

73	(¬ GSK3) → CYC1	1	[125]
74	HPK1 → MEKK1	1	[126]
75	HPK1 → MLK3	1	[127]
76	(¬ IKKAB) → IKB	1	[27]
77	IL2 → CCBL	1	[50]
78	IL2 → CrkL	1	[50]
79	IL2 → DGKa	1	[98, 99, 100, 128]
80	IL2 → PLD	1	[95, 99, 129]
81	IL2 → SHP1	1	[130]
82	IL2 ∧ IL2Ra ∧ IL2Rbg → IL2Rabg	1	receptor complex
83	IL2 ∧ IL2Rb ∧ LCKR → LCK	1	[131, 159, 160, 161]
84	IL2 ∧ LCK → LAT	1	own result
85	IL2 ∨ LCK → STAT5	1	[131]
86	IL2Rabg → Aiolos	1	[18, 134, 135, 136, 137, 138]
87	IL2Rabg → FYN	1	[39]
88	IL2Rabg ∧ JAK1 ∧ (¬ SHP1) ∧ (¬ SHP2) ∧ (¬ SOCS-1) ∧ (¬ SOCS-3) → pIL2R	1	[77, 130, 139, 140, 141, 142, 143, 144]
89	IL2Rabg ∧ (¬ SHP1) → JAK3	1	[73, 130, 131, 140, 145, 146, 147, 148]
90	IL2Rb ∧ IL2Rgc → IL2Rbg	1	receptor complex
91	IL2Rb ∧ JAK3 → Syk	1	[131, 132, 133]
92	IP3 → CA	1	[149]
93	JAK1 → IRS	1	[150]
94	JAK1 → SOCS-3	1	[141, 151]
95	JAK1 → STAT3	1	[71, 72, 73, 139, 152, 153]
96	JAK1 ∧ pIL2R → Shc	1	[103, 121, 139, 154, 155, 156, 157, 158]
97	JAK3 → IRS	1	[150]
98	JAK3 ∧ pIL2R → FYN	1	[39]
99	JAK3 ∧ pIL2R → LCK	1	[159, 160, 161]
100	JAK3 ∧ (¬ SHP1) ∧ (¬ SOCS-3) → JAK1	1	[74, 130, 131, 141]
101	JNK → JUN	1	[101]
102	LAT → GADS	1	[113, 114]
103	LAT → GRB2	1	[114, 162]

104	LAT → HPK1	1	[163]
105	LAT → PLCGB	1	[113, 114]
106	LAT ∧ ZAP70 → SH3BP2	1	[164]
107	LCK → STAT3	1	[131]
108	LCKP1 → ABL	1	[16]
109	LCKP1 → RLK	1	[165]
110	LCKP1 ∨ LCKP2 → LCK	1	pool relation
111	LCKP1 ∧ TCRB → TCRP	1	[67]
112	LCKR ∧ TCRB → FYN	1	[110, 166]
113	LCKR ∧ TCRB → LCKP2	1	[167]
114	(¬ MALT1) → A20	1	[10]
115	MEK ∨ nPKC ∨ PI3K → ERK	1	[27, 121, 168, 169, 170]
116	MEKK1 → JNK	1	[171]
117	MEKK1 → MKK4	1	[172]
118	MEKK1 → P38	1	[173]
119	MKK4 → JNK	1	[171, 174]
120	MLK3 → MKK4	1	[127]
121	mTOR → cyclin A	1	[81]
122	mTOR → (¬ P27Kip)	1	[175]
123	mTOR → P70S6K	1	[40, 168, 176, 177, 178, 179]
124	P70S6K → S6	1	[180]
125	PA → c-myc ∧ c-RAF ∧ cyclin D3 ∧ FOS	1	[128]
126	PAG → CSK	1	[114, 162]
127	PDK1 → P70S6K	1	[181, 182]
128	PDK1 → PKB	1	[168, 180, 183, 184]
129	PI3K → DGKa	1	[98, 99, 100, 128, 185]
130	PI3K → PDK1	1	[184, 186]
131	PI3K ∧ (¬ PTEN) ∧ (¬ SHIP1) → PIP3	1	[40, 46, 187, 188]
132	pIL2R → JNK	1	[156]
133	pIL2R → P38	1	[156, 170, 189, 190]
134	PIP3 → PDK1	1	[186]
135	PIP3 → PKCz	1	[88, 89, 91, 94, 96, 191, 95]
136	PIP3 ∧ SLP76 ∧ ZAP70 → ITK	1	[27, 59, 113]
137	PKB → Aiolos	1	[47]
138	(¬ PKB) → BAD	1	[192]
139	(¬ PKB) → FKHR	1	[192]
140	(¬ PKB) → GSK3	1	[125, 192]
141	PKB → mTOR	1	[193, 194, 195, 196, 197]

142	(¬ PKB) → P21Cip1	1	[125, 192]
143	(¬ PKB) → P27Kip	1	[125, 192]
144	PKCTH → nPKC	1	isoform relation
145	PLA → DAG (PLA)	1	[95, 198]
146	PLCGA → IP3	1	[27, 113]
147	PLD → DAG (PLD)	1	[88, 95, 198]
148	pRB → E2F	1	[179]
149	RAC1P1 → MLK3	1	[199]
150	RAC1P2 → MEKK1	1	[68, 69, 200]
151	RAC1P2 → SRE	1	[70]
152	RAC1R ∧ VAV1 → RAC1P1	1	[201]
153	RAC1R ∧ VAV3 → RAC1P2	1	[201]
154	RAF → MEK	1	[121, 202]
155	RAS → RAF	1	
156	RIP1 → TRAF2	1	[15]
157	RIP2 → TRAF6	1	[1]
158	RSK → CREB	1	[102]
159	S6 → cyclin D3	1	[179]
160	SH3BP2 → VAV3	1	[201]
161	SH3BP2 ∧ ZAP70 → VAV1	1	[164, 201]
162	Shc → GRB2	1	[121, 124, 156, 155, 203, 204, 205]
163	STAT3 ∨ STAT5 → Blimp-1	1	[206, 207]
164	Syk → STAT3	1	[131]
165	Syk → STAT5	1	[131]
166	TCRB → DGK	2	[208]
167	X → VAV1	1	[63, 64, 65, 209]
168	ZAP70 → LAT	1	[27]

References

[1] Sun L, Deng L, Ea CK, Xia ZP, Chen ZJ: **The TRAF6 ubiquitin ligase and TAK1 kinase mediate IKK activation by BCL10 and MALT1 in T lymphocytes.** Mol Cell 2004, **14**:289-301.

[2] Uren AG, O'Rourke K, Aravind LA, Pisabarro MT, Seshagiri S, Koonin EV, Dixit VM: **Identification of paracaspases and metacaspases: two ancient families of caspase-like proteins, one of which plays a key role in MALT lymphoma.** Mol Cell 2000, **6**:961-7.

[3] Lucas PC, Yonezumi M, Inohara N, McAllister-Lucas LM, Abazeed ME, Chen FF, Yamaoka S, Seto M, Nunez G: **Bcl10 and MALT1, independent targets of chromosomal translocation in malt lymphoma, cooperate in a novel NF-kappa B signaling pathway.** J Biol Chem 2001, **276**:19012-9.

[4] Gaide O, Favier B, Legler DF, Bonnet D, Brissoni B, Valitutti S, Bron C, Tschopp J, Thome M: **CARMA1 is a critical lipid raft-associated regulator of TCR-induced NF-kappa B activation.** Nat Immunol 2002, **3**:836-43.

[5] Thome M: **CARMA1, BCL-10 and MALT1 in lymphocyte development and activation.** Nat Rev Immunol 2004, **4**:348-59.

[6] Hara H, Bakal C, Wada T, Bouchard D, Rottapel R, Saito T, Penninger JM: **The molecular adapter Carma1 controls entry of IkappaB kinase into the central immune synapse.** J Exp Med 2004, **200**:1167-77.

[7] Sommer K, Guo B, Pomerantz JL, Bandaranayake AD, Moreno-García ME, Ovechkina YL, Rawlings DJ: **Phosphorylation of the CARMA1 linker controls NF-kappaB activation.** Immunity 2005, **23**:561-74.

[8] Matsumoto R, Wang D, Blonska M, Li H, Kobayashi M, Pappu B, Chen Y, Wang D, Lin X: **Phosphorylation of CARMA1 plays a critical role in T Cell receptor-mediated NF-kappaB activation.** Immunity 2005, **23**:575-85.

[9] Oeckinghaus A, Wegener E, Welteke V, Ferch U, Arslan SC, Ruland J, Scheidereit C, Krappmann D: **Malt1 ubiquitination triggers NF-kappaB signaling upon T-cell activation.** EMBO J 2007, **26**:4634-45.

[10] Coornaert B, Baens M, Heyninck K, Bekaert T, Haegman M, Staal J, Sun L, Chen ZJ, Marynen P, Beyaert R: **T cell antigen receptor stimulation induces MALT1 paracaspase-mediated cleavage of the NF-kappaB inhibitor A20.** Nat Immunol 2008, **9**:263-71.

[11] Stilo R, Varricchio E, Liguoro D, Leonardi A, Vito P: **A20 is a negative regulator of BCL10- and CARMA3-mediated activation of NF-kappaB.** J Cell Sci 2008, **121**:1165-71.

[12] Ruefli-Brasse AA, Lee WP, Hurst S, Dixit VM: **Rip2 participates in Bcl10 signaling and T-cell receptor-mediated NF-kappaB activation.** J Biol Chem 2004, **279**:1570-4.

[13] Tewari M, Wolf FW, Seldin MF, O'Shea KS, Dixit VM, Turka LA: **Lymphoid expression and regulation of A20, an inhibitor of programmed cell death.** J Immunol 1995, **154**:1699-706.

[14] Dohrman A, Russell JQ, Cuenin S, Fortner K, Tschopp J, Budd RC: **Cellular FLIP long form augments caspase activity and death of T cells through heterodimerization with and activation of caspase-8.** J Immunol 2005, **175**:311-8.

[15] Misra RS, Russell JQ, Koenig A, Hinshaw-Makepeace JA, Wen R, Wang D, Huo H, Littman DR, Ferch U, Ruland J, Thome M, Budd RC: **Caspase-8 and c-FLIPL associate in lipid rafts with NF-kappaB adaptors during T cell activation.** J Biol Chem 2007, **282**:19365-74.

[16] Zipfel PA, Zhang W, Quiroz M, Pendergast AM: **Requirement for Abl kinases in T cell receptor signaling.** Curr Biol 2004, **14**:1222-31.

[17] Miyazaki T, Liu ZJ, Kawahara A, Minami Y, Yamada K, Tsujimoto Y, Barsoumian EL, Permutter RM, Taniguchi T: **Three distinct IL-2 signaling pathways mediated by bcl-2, c-myc, and lck cooperate in hematopoietic cell proliferation.** Cell 1995, **81**:223-31.

[18] Romero F, Martínez-A C, Camonis J, Rebollo A: **Aiolos transcription factor controls cell death in T cells by regulating Bcl-2 expression and its cellular localization.** EMBO J 1999, **18**:3419-30.

[19] Moon JJ, Nelson BH: **Phosphatidylinositol 3-kinase potentiates, but does not trigger, T cell proliferation mediated by the IL-2 receptor.** J Immunol 2001, **167**:2714-23.

[20] Kashishian A, Howard M, Loh C, Gallatin WM, Hoekstra MF, Lai Y: **AKAP79 inhibits calcineurin through a site distinct from the immunophilin-binding region.** J Biol Chem 1998, **273**:27412-9.

[21] Matsuda S, Shibasaki F, Takehana K, Mori H, Nishida E, Koyasu S: **Two distinct action mechanisms of immunophilin-ligand complexes for the blockade of T-cell activation.** EMBO Rep 2000, **1**:428-34.

[22] Ryeom S, Greenwald RJ, Sharpe AH, McKeon F: **The threshold pattern of calcineurin-dependent gene expression is altered by loss of the endogenous inhibitor calcipressin.** Nat Immunol 2003, **4**:874-81.

[23] Yang E, Zha J, Jockel J, Boise LH, Thompson CB, Korsmeyer SJ: **Bad, a heterodimeric partner for Bcl-XL and Bcl-2, displaces Bax and promotes cell death.** Cell 1995, **80**:285-91.

[24] Zha J, Harada H, Osipov K, Jockel J, Waksman G, Korsmeyer SJ: **BH3 domain of BAD is required for heterodimerization with BCL-XL and pro-apoptotic activity.** J Biol Chem 1997, **272**:24101-4.

[25] Bidère N, Snow AL, Sakai K, Zheng L, Lenardo MJ: **Caspase-8 regulation by direct interaction with TRAF6 in T cell receptor-induced NF-kappaB activation.** Curr Biol 2006 **16**:1666-71.

[26] McCully RR, Pomerantz JL: **The protein kinase C-responsive inhibitory domain of CARD11 functions in NF-kappaB activation to regulate the association of multiple signaling cofactors that differentially depend on Bcl10 and MALT1 for association.** Mol Cell Biol 2008 **28**:5668-86.

[27] Huang Y, Wange RL: **T cell receptor signaling: beyond complex complexes.** J Biol Chem 2004, **279**:28827-30.

[28] Round JL, Tomassian T, Zhang M, Patel V, Schoenberger SP, Miceli MC: **Dlgh1 coordinates actin polymerization, synaptic T cell receptor and lipid raft aggregation, and effector function in T cells.** J Exp Med 2005, **201**:419-30.

[29] Macian F: **NFAT proteins: key regulators of T-cell development and function.** Nat Rev Immunol 2005, **5**:472-84.

[30] Round JL, Humphries LA, Tomassian T, Mittelstadt P, Zhang M, Miceli MC: **Scaffold protein Dlgh1 coordinates alternative p38 kinase activation, directing T cell receptor signals toward NFAT but not NF-kappaB transcription factors.** Nat Immunol 2007, **8**:154-61.

[31] Gong D, Malek TR: **Cytokine-dependent Blimp-1 expression in activated T cells inhibits IL-2 production.** J Immunol 2007, **178**:242-52.

[32] Feske S, Giltnane J, Dolmetsch R, Staudt LM, Rao A: **Gene regulation mediated by calcium signals in T lymphocytes.** Nat Immunol 2001, **2**:316-24.

[33] Hughes K, Edin S, Antonsson A, Grundström T: **Calmodulin-dependent kinase II mediates T cell receptor/CD3- and phorbol ester-induced activation of IkappaB kinase.** J Biol Chem 2001, **276**:36008-13.

[34] Anderson KA, Kane CD: **Ca2+/calmodulin-dependent protein kinase IV and calcium signaling.** Biometals 1998, **11**:331-43.

[35] Hayden MS, Ghosh S: **Signaling to NF-kappaB.** Genes Dev 2004, **18**:2195-224.

[36] Weil R, Israël A: **Deciphering the pathway from the TCR to NF-kappaB.** Cell Death Differ 2006, **13**:826-33.

[37] Pan F, Means AR, Liu JO: **Calmodulin-dependent protein kinase IV regulates nuclear export of Cabin1 during T-cell activation.** EMBO J 2005, **24**:2104-13.

[38] Krauss G; **Biochemistry of Signal Transduction and Regulation.** Wiley-VCH 2003.

[39] Karnitz LM, Sutor SL, Abraham RT: **The Src-family kinase, Fyn, regulates the activation of phosphatidylinositol 3-kinase in an interleukin 2-responsive T cell line.** J Exp Med 1994, **179**:1799-808.

[40] Monfar M, Lemon KP, Grammer TC, Cheatham L, Chung J, Vlahos CJ, Blenis J: **Activation of pp70/85 S6 kinases in interleukin-2-responsive lymphoid cells is mediated by phosphatidylinositol 3-kinase and inhibited by cyclic AMP.** Mol Cell Biol 1995, **15**:326-37.

[41] Augustine JA, Sutor SL, Abraham RT: **Interleukin 2- and polyomavirus middle T antigen-induced modification of phosphatidylinositol 3-kinase activity in activated T lymphocytes.** Mol Cell Biol 1991, **11**:4431-40.

[42] Merida I, Diez E, Gaulton GN: **IL-2 binding activates a tyrosine-phosphorylated phosphatidylinositol-3-kinase.** J Immunol 1991, **147**:2202-7.

[43] Remillard B, Petrillo R, Maslinski W, Tsudo M, Strom TB, Cantley L, Varticovski L: **Interleukin-2 receptor regulates activation of phosphatidylinositol 3-kinase.** J Biol Chem 1991, **266**:14167-70.

[44] Truitt KE, Mills GB, Turck CW, Imboden JB: **SH2-dependent association of phosphatidylinositol 3’-kinase 85-kDa regulatory subunit with the interleukin-2 receptor beta chain.** J Biol Chem 1994, **269**:5937-43.

[45] Kanazawa T, Keeler ML, Varticovski L: **Serine-rich region of the IL-2 receptor beta-chain is required for activation of phosphatidylinositol 3-kinase.** Cell Immunol 1994, **156**:378-88.

[46] Taichman R, Merida I, Torigoe T, Gaulton GN, Reed JC: **Evidence that protein tyrosine kinase p56-Lck regulates the activity of phosphatidylinositol-3’-kinase in interleukin-2-dependent T-cells.** J Biol Chem 1993, **268**:20031-6.

[47] Ahmed NN, Grimes HL, Bellacosa A, Chan TO, Tsichlis PN: **Transduction of interleukin-2 antiapoptotic and proliferative signals via Akt protein kinase.** Proc Natl Acad Sci U S A 1997, **94**:3627-32.

[48] Gesbert F, Guenzi C, Bertoglio J: **A new tyrosine-phosphorylated 97-kDa adaptor protein mediates interleukin-2-induced association of SHP-2 with p85-phosphatidylinositol 3-kinase in human T lymphocytes.** J Biol Chem 1998, **273**:18273-81.

[49] Crouin C, Arnaud M, Gesbert F, Camonis J, Bertoglio J: **A yeast two-hybrid study of human p97/Gab2 interactions with its SH2 domain-containing binding partners.** FEBS Lett 2001, **495**:148-53.

[50] Gesbert F, Garbay C, Bertoglio J: **Interleukin-2 stimulation induces tyrosine phosphorylation of p120-Cbl and CrkL and formation of multimolecular signaling complexes in T lymphocytes and natural killer cells.** J Biol Chem 1998, **273**:3986-93.

[51] Fang D, Liu YC: **Proteolysis-independent regulation of PI3K by Cbl-b-mediated ubiquitination in T cells.** Nat Immunol 2001, **2**:870-5.

[52] Deane JA, Fruman DA: **Phosphoinositide 3-kinase: diverse roles in immune cell activation.** Annu Rev Immunol 2004, **22**:563-98.

[53] von Willebrand M, Baier G, Couture C, Burn P, Mustelin T: **Activation of phosphatidylinositol-3-kinase in Jurkat T cells depends on the presence of the p56lck tyrosine kinase.** Eur J Immunol 1994, **24**:234-8.

[54] August A, Dupont B: **CD28 of T lymphocytes associates with phosphatidylinositol 3-kinase.** Int Immunol 1994, **6**:769-74.

[55] Ghiotto-Ragueneau M, Battifora M, Truneh A, Waterfield MD, Olive D: **Comparison of CD28-B7.1 and B7.2 functional interaction in resting human T cells: phosphatidylinositol 3-kinase association to CD28 and cytokine production.** Eur J Immunol 1996, **26**:34-41.

[56] Gibson S, Truitt K, Lu Y, Lapushin R, Khan H, Imboden JB, Mills GB: **Efficient CD28 signalling leads to increases in the kinase activities of the TEC family tyrosine kinase EMT/ITK/TSK and the SRC family tyrosine kinase LCK.** Biochem J 1998, **330 (Pt 3)**:1123-8.

[57] Holdorf AD, Green JM, Levin SD, Denny MF, Straus DB, Link V, Changelian PS, Allen PM, Shaw AS: **Proline residues in CD28 and the Src homology (SH)3 domain of Lck are required for T cell costimulation.** J Exp Med 1999, **190**:375-84.

[58] Huang J, Lo PF, Zal T, Gascoigne NR, Smith BA, Levin SD, Grey HM: **CD28 plays a critical role in the segregation of PKC theta within the immunologic synapse.** Proc Natl Acad Sci U S A 2002, **99**:9369-73.

[59] Czar MJ, Debnath J, Schaeffer EM, Lewis CM, Schwartzberg PL: **Biochemical and genetic analyses of the Tec kinases Itk and Rlk/Txk.** Biochem Soc Trans 2001, **29**:863-7.

[60] Rellahan BL, Graham LJ, Tysgankov AY, DeBell KE, Veri MC, Noviello C, Bonvini E: **A dynamic constitutive and inducible binding of c-Cbl by PLCgamma1 SH3 and SH2 domains (negatively) regulates antigen receptor-induced PLCgamma1 activation in lymphocytes.** Exp Cell Res 2003, **289**:184-94.

[61] Rao N, Lupher ML, Ota S, Reedquist KA, Druker BJ, Band H: **The linker phosphorylation site Tyr292 mediates the negative regulatory effect of Cbl on ZAP-70 in T cells.** J Immunol 2000, **164**:4616-26.

[62] Zhang J, Bárdos T, Li D, Gál I, Vermes C, Xu J, Mikecz K, Finnegan A, Lipkowitz S, Glant TT: **Cutting edge: regulation of T cell activation threshold by CD28 costimulation through targeting Cbl-b for ubiquitination.** J Immunol 2002, **169**:2236-40.

[63] Michel F, Mangino G, Attal-Bonnefoy G, Tuosto L, Alcover A, Roumier A, Olive D, Acuto O: **CD28 utilizes Vav-1 to enhance TCR-proximal signaling and NF-AT activation.** J Immunol 2000, **165**:3820-9.

[64] Raab M, Pfister S, Rudd CE: **CD28 signaling via VAV/SLP-76 adaptors: regulation of cytokine transcription independent of TCR ligation.** Immunity 2001, **15**:921-33.

[65] Kovacs B, Parry RV, Ma Z, Fan E, Shivers DK, Freiberg BA, Thomas AK, Rutherford R, Rumbley CA, Riley JL, Finkel TH: **Ligation of CD28 by its natural ligand CD86 in the absence of TCR stimulation induces lipid raft polarization in human CD4 T cells.** J Immunol 2005, **175**:7848-54.

[66] Palacios EH, Weiss A: **Function of the Src-family kinases, Lck and Fyn, in T-cell development and activation.** Oncogene 2004, **23**:7990-8000.

[67] Filipp D, Leung BL, Zhang J, Veillette A, Julius M: **Enrichment of lck in lipid rafts regulates colocalized fyn activation and the initiation of proximal signals through TCR alpha beta.** J Immunol 2004, **172**:4266-74.

[68] Fanger GR, Johnson NL, Johnson GL: **MEK kinases are regulated by EGF and selectively interact with Rac/Cdc42.** EMBO J 1997, **16**:4961-72.

[69] Kaga S, Ragg S, Rogers KA, Ochi A: **Activation of p21-CDC42/Rac-activated kinases by CD28 signaling: p21-activated kinase (PAK) and MEK kinase 1 (MEKK1) may mediate the interplay between CD3 and CD28 signals.** J Immunol 1998, **160**:4182-9.

[70] Hill CS, Wynne J, Treisman R: **The Rho family GTPases RhoA, Rac1, and CDC42Hs regulate transcriptional activation by SRF.** Cell 1995, **81**:1159-70.

[71] Johnston JA, Bacon CM, Finbloom DS, Rees RC, Kaplan D, Shibuya K, Ortaldo JR, Gupta S, Chen YQ, Giri JD: **Tyrosine phosphorylation and activation of STAT5, STAT3, and Janus kinases by interleukins 2 and 15.** Proc Natl Acad Sci U S A 1995, **92**:8705-9.

[72] Hou J, Schindler U, Henzel WJ, Wong SC, McKnight SL: **Identification and purification of human Stat proteins activated in response to interleukin-2.** Immunity 1995, **2**:321-9.

[73] Beadling C, Guschin D, Witthuhn BA, Ziemiecki A, Ihle JN, Kerr IM, Cantrell DA: **Activation of JAK kinases and STAT proteins by interleukin-2 and interferon alpha, but not the T cell antigen receptor, in human T lymphocytes.** EMBO J 1994, **13**:5605-15.

[74] Liu KD, Gaffen SL, Goldsmith MA, Greene WC: **Janus kinases in interleukin-2-mediated signaling: JAK1 and JAK3 are differentially regulated by tyrosine phosphorylation.** Curr Biol 1997, **7**:817-26.

[75] Matsumoto A, Seki Y, Kubo M, Ohtsuka S, Suzuki A, Hayashi I, Tsuji K, Nakahata T, Okabe M, Yamada S, Yoshimura A: **Suppression of STAT5 functions in liver, mammary glands, and T cells in cytokine-inducible SH2-containing protein 1 transgenic mice.** Mol Cell Biol 1999, **19**:6396-407.

[76] Aman MJ, Migone TS, Sasaki A, Ascherman DP, Zhu M, Soldaini E, Imada K, Miyajima A, Yoshimura A, Leonard WJ: **CIS associates with the interleukin-2 receptor beta chain and inhibits interleukin-2-dependent signaling.** J Biol Chem 1999, **274**:30266-72.

[77] Cornish AL, Chong MM, Davey GM, Darwiche R, Nicola NA, Hilton DJ, Kay TW, Starr R, Alexander WS: **Suppressor of cytokine signaling-1 regulates signaling in response to interleukin-2 and other gamma c-dependent cytokines in peripheral T cells.** J Biol Chem 2003, **278**:22755-61.

[78] Arnaud M, Mzali R, Gesbert F, Crouin C, Guenzi C, Vermot-Desroches C, Wijdenes J, Courtois G, Bernard O, Bertoglio J: **Interaction of the tyrosine phosphatase SHP-2 with Gab2 regulates Rho-dependent activation of the c-fos serum response element by interleukin-2.** Biochem J 2004, **382**:545-56.

[79] Flanagan WM, Crabtree GR: **Rapamycin inhibits p34cdc2 expression and arrests T lymphocyte proliferation at the G1/S transition.** Ann N Y Acad Sci 1993, **696**:31-7.

[80] Morice WG, Wiederrecht G, Brunn GJ, Siekierka JJ, Abraham RT: **Rapamycin inhibition of interleukin-2-dependent p33cdk2 and p34cdc2 kinase activation in T lymphocytes.** J Biol Chem 1993, **268**:22737-45.

[81] Morice WG, Brunn GJ, Wiederrecht G, Siekierka JJ, Abraham RT: **Rapamycin-induced inhibition of p34cdc2 kinase activation is associated with G1/S-phase growth arrest in T lymphocytes.** J Biol Chem 1993, **268**:3734-8.

[82] Bivona TG, Pérez De Castro I, Ahearn IM, Grana TM, Chiu VK, Lockyer PJ, Cullen PJ, Pellicer A, Cox AD, Philips MR: **Phospholipase Cgamma activates Ras on the Golgi apparatus by means of RasGRP1.** Nature 2003, **424**:694-8.

[83] Di Fiore PP: **Signal transduction: life on Mars, cellularly speaking.** Nature 2003, **424**:624-5.

[84] Roose JP, Mollenauer M, Gupta VA, Stone J, Weiss A: **A diacylglycerol-protein kinase C-RasGRP1 pathway directs Ras activation upon antigen receptor stimulation of T cells.** Mol Cell Biol 2005, **25**:4426-41.

[85] Villalba M, Bi K, Hu J, Altman Y, Bushway P, Reits E, Neefjes J, Baier G, Abraham RT, Altman A: **Translocation of PKC[theta] in T cells is mediated by a nonconventional, PI3-K- and Vav-dependent pathway, but does not absolutely require phospholipase C.** J Cell Biol 2002, **157**:253-63.

[86] Lee KY, D'Acquisto F, Hayden MS, Shim JH, Ghosh S: **PDK1 nucleates T cell receptor-induced signaling complex for NF-kappaB activation.** Science 2005, **308**:114-8.

[87] Gruber T, Freeley M, Thuille N, Heit I, Shaw S, Long A, Baier G: **Comment on "PDK1 nucleates T cell receptor-induced signaling complex for NF-kappaB activation".** Science 2006, **312**:55; author reply 55.

[88] Sawami H, Terada N, Franklin RA, Okawa H, Uchiyama T, Lucas JJ, Gelfand EW: **Signal transduction by interleukin 2 in human T cells: activation of tyrosine and ribosomal S6 kinases and cell-cycle regulatory genes.** J Cell Physiol 1992, **151**:367-77.

[89] Clark RB, Love JT, Sgroi D, Lingenheld EG, Sha'afi RI: **The protein kinase C inhibitor H-7, inhibits antigen and IL-2-induced proliferation of murine T cell lines.** Biochem Biophys Res Commun 1987, **145**:666-72.

[90] Fong TC, Makinodan T: **Preferential enhancement by 2-mercaptoethanol of IL-2 responsiveness of T blast cells from old over young mice is associated with potentiated protein kinase C translocation.** Immunol Lett 1989, **20**:149-54.

[91] Redondo JM, López-Rivas A, Vila V, Cragoe EJ, Fresno M: **The role of protein kinase C in T lymphocyte proliferation. Existence of protein kinase C-dependent and -independent pathways.** J Biol Chem 1988, **263**:17467-70.

[92] Mahé Y, Piau JP, Wakasugi N, Tursz T, Gacon G, Wakasugi H: **Comparison of interleukin 2 and 12-O-tetradecanoylphorbol 13-acetate as signals for protein kinase C activation in purified human T lymphocytes.** Eur J Immunol 1988, **18**:2029-36.

[93] Farrar WL, Anderson WB: **Interleukin-2 stimulates association of protein kinase C with plasma membrane.** Nature , **315**:233-5.

[94] Valge VE, Wong JG, Datlof BM, Sinskey AJ, Rao A: **Protein kinase C is required for responses to T cell receptor ligands but not to interleukin-2 in T cells.** Cell 1988, **55**:101-12.

[95] Lu Y, Morley P, Durkin JP: **Signalling events mediating the activation of protein kinase C by interleukin-2 in cytotoxic T cells.** Cell Signal 1999, **11**:275-85.

[96] Gómez J, Pitton C, García A, Martinez de Aragón A, Silva A, Rebollo A: **The zeta isoform of protein kinase C controls interleukin-2-mediated proliferation in a murine T cell line: evidence for an additional role of protein kinase C epsilon and beta.** Exp Cell Res 1995, **218**:105-13.

[97] Topham MK: **Signaling roles of diacylglycerol kinases.** J Cell Biochem 2006, **97**:474-84.

[98] Flores I, Casaseca T, Martinez-A C, Kanoh H, Merida I: **Phosphatidic acid generation through interleukin 2 (IL-2)-induced alpha-diacylglycerol kinase activation is an essential step in IL-2-mediated lymphocyte proliferation.** J Biol Chem 1996, **271**:10334-40.

[99] Jones DR, Flores I, Díaz E, Martinez C, Mérida I: **Interleukin-2 stimulates a late increase in phosphatidic acid production in the absence of phospholipase D activation.** FEBS Lett 1998, **433**:23-7.

[100] Jones DR, Pettitt TR, Sanjuán MA, Mérida I, Wakelam MJ: **Interleukin-2 causes an increase in saturated/monounsaturated phosphatidic acid derived from 1,2-diacylglycerol and 1-O-alkyl-2-acylglycerol.** J Biol Chem 1999, **274**:16846-52.

[101] Satoh T, Minami Y, Kono T, Yamada K, Kawahara A, Taniguchi T, Kaziro Y: **Interleukin 2-induced activation of Ras requires two domains of interleukin 2 receptor beta subunit, the essential region for growth stimulation and Lck-binding domain.** J Biol Chem 1992, **267**:25423-7.

[102] Frödin M, Gammeltoft S: **Role and regulation of 90 kDa ribosomal S6 kinase (RSK) in signal transduction.** Mol Cell Endocrinol 1999, **151**:65-77.

[103] Gadina M, Sudarshan C, Visconti R, Zhou YJ, Gu H, Neel BG, O'Shea JJ: **The docking molecule gab2 is induced by lymphocyte activation and is involved in signaling by interleukin-2 and interleukin-15 but not other common gamma chain-using cytokines.** J Biol Chem 2000, **275**:26959-66.

[104] Brockdorff JL, Gu H, Mustelin T, Kaltoft K, Geisler C, Röpke C, Ødum N: **Gab2 is phosphorylated on tyrosine upon interleukin-2/interleukin-15 stimulation in mycosis-fungoides-derived tumor T cells and associates inducibly with SHP-2 and Stat5a.** Exp Clin Immunogenet 2001, **18**:86-95.

[105] Arnaud M, Crouin C, Deon C, Loyaux D, Bertoglio J: **Phosphorylation of Grb2-associated binder 2 on serine 623 by ERK MAPK regulates its association with the phosphatase SHP-2 and decreases STAT5 activation.** J Immunol 2004, **173**:3962-71.

[106] Stefanová I, Dorfman JR, Germain RN: **Self-recognition promotes the foreign antigen sensitivity of naive T lymphocytes.** Nature 2002, **420**:429-34.

[107] Altan-Bonnet G, Germain RN: **Modeling T cell antigen discrimination based on feedback control of digital ERK responses.** PLoS Biol 2005, **3**:e356.

[108] Torgersen KM, Vang T, Abrahamsen H, Yaqub S, Horejsí V, Schraven B, Rolstad B, Mustelin T, Taskén K: **Release from tonic inhibition of T cell activation through transient displacement of C-terminal Src kinase (Csk) from lipid rafts.** J Biol Chem 2001, **276**:29313-8.

[109] Davidson D, Bakinowski M, Thomas ML, Horejsi V, Veillette A: **Phosphorylation-dependent regulation of T-cell activation by PAG/Cbp, a lipid raft-associated transmembrane adaptor.** Mol Cell Biol 2003, **23**:2017-28.

[110] Filipp D, Julius M: **Lipid rafts: resolution of the ”fyn problem”?** Mol Immunol 2004, **41**:645-56.

[111] Yamasaki S, Nishida K, Hibi M, Sakuma M, Shiina R, Takeuchi A, Ohnishi H, Hirano T, Saito T: **Docking protein Gab2 is phosphorylated by ZAP-70 and negatively regulates T cell receptor signaling by recruitment of inhibitory molecules.** J Biol Chem 2001, **276**:45175-83.

[112] Yamasaki S, Nishida K, Sakuma M, Berry D, McGlade CJ, Hirano T, Saito T: **Gads/Grb2-mediated association with LAT is critical for the inhibitory function of Gab2 in T cells.** Mol Cell Biol 2003, **23**:2515-29.

[113] Togni M, Lindquist J, Gerber A, Kölsch U, Hamm-Baarke A, Kliche S, Schraven B: **The role of adaptor proteins in lymphocyte activation.** Mol Immunol 2004, **41**:615-30.

[114] Horejsí V, Zhang W, Schraven B: **Transmembrane adaptor proteins: organizers of immunoreceptor signalling.** Nat Rev Immunol 2004, **4**:603-16.

[115] Salvador JM, Mittelstadt PR, Guszczynski T, Copeland TD, Yamaguchi H, Appella E, Fornace AJ, Ashwell JD: **Alternative p38 activation pathway mediated by T cell receptor-proximal tyrosine kinases.** Nat Immunol 2005, **6**:390-5.

[116] Salvador JM, Mittelstadt PR, Belova GI, Fornace AJ, Ashwell JD: **The autoimmune suppressor Gadd45alpha inhibits the T cell alternative p38 activation pathway.** Nat Immunol 2005, **6**:396-402.

[117] Jirmanova L, Jankovic D, Fornace AJ, Ashwell JD: **Gadd45alpha regulates p38-dependent dendritic cell cytokine production and Th1 differentiation.** J Immunol 2007, **178**:4153-8.

[118] Genot E, Cantrell DA: **Ras regulation and function in lymphocytes.** Curr Opin Immunol 2000, **12**:289-94.

[119] Graves JD, Downward J, Izquierdo-Pastor M, Rayter S, Warne PH, Cantrell DA: **The growth factor IL-2 activates p21ras proteins in normal human T lymphocytes.** J Immunol 1992, **148**:2417-22.

[120] Satoh T, Nakafuku M, Miyajima A, Kaziro Y: **Involvement of ras p21 protein in signal-transduction pathways from interleukin 2, interleukin 3, and granulocyte/macrophage colony-stimulating factor, but not from interleukin 4.** Proc Natl Acad Sci U S A 1991, **88**:3314-8.

[121] Karnitz LM, Burns LA, Sutor SL, Blenis J, Abraham RT: **Interleukin-2 triggers a novel phosphatidylinositol 3-kinase-dependent MEK activation pathway.** Mol Cell Biol 1995, **15**:3049-57.

[122] Buday L, Egan SE, Rodriguez Viciana P, Cantrell DA, Downward J: **A complex of Grb2 adaptor protein, Sos exchange factor, and a 36-kDa membrane-bound tyrosine phosphoprotein is implicated in ras activation in T cells.** J Biol Chem 1994, **269**:9019-23.

[123] Nishida K, Yoshida Y, Itoh M, Fukada T, Ohtani T, Shirogane T, Atsumi T, Takahashi-Tezuka M, Ishihara K, Hibi M, Hirano T: **Gab-family adapter proteins act downstream of cytokine and growth factor receptors and T- and B-cell antigen receptors.** Blood 1999, **93**:1809-16.

[124] Gu H, Maeda H, Moon JJ, Lord JD, Yoakim M, Nelson BH, Neel BG: **New role for Shc in activation of the phosphatidylinositol 3-kinase/Akt pathway.** Mol Cell Biol 2000, **20**:7109-20.

[125] Liang J, Slingerland JM: **Multiple roles of the PI3K/PKB (Akt) pathway in cell cycle progression.** Cell Cycle , **2**:339-45.

[126] Hu MC, Qiu WR, Wang X, Meyer CF, Tan TH: **Human HPK1, a novel human hematopoietic progenitor kinase that activates the JNK/SAPK kinase cascade.** Genes Dev 1996, **10**:2251-64.

[127] Tibbles LA, Ing YL, Kiefer F, Chan J, Iscove N, Woodgett JR, Lassam NJ: **MLK-3 activates the SAPK/JNK and p38/RK pathways via SEK1 and MKK3/6.** EMBO J 1996, **15**:7026-35.

[128] Flores I, Jones DR, Ciprés A, Díaz-Flores E, Sanjuan MA, Mérida I: **Diacylglycerol kinase inhibition prevents IL-2-induced G1 to S transition through a phosphatidylinositol-3 kinase-independent mechanism.** J Immunol 1999, **163**:708-14.

[129] Cano E, Muñoz-Fernández MA, Fresno M: **Regulation of interleukin-2 responses by phosphatidic acid.** Eur J Immunol 1992, **22**:1883-9.

[130] Migone TS, Cacalano NA, Taylor N, Yi T, Waldmann TA, Johnston JA: **Recruitment of SH2-containing protein tyrosine phosphatase SHP-1 to the interleukin 2 receptor; loss of SHP-1 expression in human T-lymphotropic virus type I-transformed T cells.** Proc Natl Acad Sci U S A 1998, **95**:3845-50.

[131] Zhou YJ, Magnuson KS, Cheng TP, Gadina M, Frucht DM, Galon J, Candotti F, Geahlen RL, Changelian PS, O'Shea JJ: **Hierarchy of protein tyrosine kinases in interleukin-2 (IL-2) signaling: activation of syk depends on Jak3; however, neither Syk nor Lck is required for IL-2-mediated STAT activation.** Mol Cell Biol 2000, **20**:4371-80.

[132] Qin S, Inazu T, Yang C, Sada K, Taniguchi T, Yamamura H: **Interleukin 2 mediates p72syk activation in peripheral blood lymphocytes.** FEBS Lett 1994, **345**:233-6.

[133] Minami Y, Nakagawa Y, Kawahara A, Miyazaki T, Sada K, Yamamura H, Taniguchi T: **Protein tyrosine kinase Syk is associated with and activated by the IL-2 receptor: possible link with the c-myc induction pathway.** Immunity 1995, **2**:89-100.

[134] Otani H, Erdos M, Leonard WJ: **Tyrosine kinase(s) regulate apoptosis and bcl-2 expression in a growth factor-dependent cell line.** J Biol Chem 1993, **268**:22733-6.

[135] Gaffen SL, Lai SY, Ha M, Liu X, Hennighausen L, Greene WC, Goldsmith MA: **Distinct tyrosine residues within the interleukin-2 receptor beta chain drive signal transduction specificity, redundancy, and diversity.** J Biol Chem 1996, **271**:21381-90.

[136] Lord JD, McIntosh BC, Greenberg PD, Nelson BH: **The IL-2 receptor promotes proliferation, bcl-2 and bcl-x induction, but not cell viability through the adapter molecule Shc.** J Immunol 1998, **161**:4627-33.

[137] Tsujino S, Di Santo JP, Takaoka A, McKernan TL, Noguchi S, Taya C, Yonekawa H, Saito T, Taniguchi T, Fujii H: **Differential requirement of the cytoplasmic subregions of gamma c chain in T cell development and function.** Proc Natl Acad Sci U S A 2000, **97**:10514-9.

[138] Lindemann MJ, Benczik M, Gaffen SL: **Anti-apoptotic signaling by the interleukin-2 receptor reveals a function for cytoplasmic tyrosine residues within the common gamma (gamma c) receptor subunit.** J Biol Chem 2003, **278**:10239-49.

[139] Friedmann MC, Migone TS, Russell SM, Leonard WJ: **Different interleukin 2 receptor beta-chain tyrosines couple to at least two signaling pathways and synergistically mediate interleukin 2-induced proliferation.** Proc Natl Acad Sci U S A 1996, **93**:2077-82.

[140] Fujii H, Ogasawara K, Otsuka H, Suzuki M, Yamamura K, Yokochi T, Miyazaki T, Suzuki H, Mak TW, Taki S, Taniguchi T: **Functional dissection of the cytoplasmic subregions of the IL-2 receptor betac chain in primary lymphocyte populations.** EMBO J 1998, **17**:6551-7.

[141] Cohney SJ, Sanden D, Cacalano NA, Yoshimura A, Mui A, Migone TS, Johnston JA: **SOCS-3 is tyrosine phosphorylated in response to interleukin-2 and suppresses STAT5 phosphorylation and lymphocyte proliferation.** Mol Cell Biol 1999, **19**:4980-8.

[142] Marine JC, Topham DJ, McKay C, Wang D, Parganas E, Stravopodis D, Yoshimura A, Ihle JN: **SOCS1 deficiency causes a lymphocyte-dependent perinatal lethality.** Cell 1999, **98**:609-16.

[143] Cacalano NA, Sanden D, Johnston JA: **Tyrosine-phosphorylated SOCS-3 inhibits STAT activation but binds to p120 RasGAP and activates Ras.** Nat Cell Biol 2001, **3**:460-5.

[144] Ilangumaran S, Ramanathan S, La Rose J, Poussier P, Rottapel R: **Suppressor of cytokine signaling 1 regulates IL-15 receptor signaling in CD8+CD44high memory T lymphocytes.** J Immunol 2003, **171**:2435-45.

[145] Johnston JA, Kawamura M, Kirken RA, Chen YQ, Blake TB, Shibuya K, Ortaldo JR, McVicar DW, O'Shea JJ: **Phosphorylation and activation of the Jak-3 Janus kinase in response to interleukin-2.** Nature 1994, **370**:151-3.

[146] Witthuhn BA, Silvennoinen O, Miura O, Lai KS, Cwik C, Liu ET, Ihle JN: **Involvement of the Jak-3 Janus kinase in signalling by interleukins 2 and 4 in lymphoid and myeloid cells.** Nature 1994, **370**:153-7.

[147] Zhou YJ, Hanson EP, Chen YQ, Magnuson K, Chen M, Swann PG, Wange RL, Changelian PS, O'Shea JJ: **Distinct tyrosine phosphorylation sites in JAK3 kinase domain positively and negatively regulate its enzymatic activity.** Proc Natl Acad Sci U S A 1997, **94**:13850-5.

[148] Cheng H, Ross JA, Frost JA, Kirken RA: **Phosphorylation of human Jak3 at tyrosines 904 and 939 positively regulates its activity.** Mol Cell Biol 2008, **28**:2271-82.

[149] Chan TO, Rittenhouse SE, Tsichlis PN: **AKT/PKB and other D3 phosphoinositide-regulated kinases: kinase activation by phosphoinositide-dependent phosphorylation.** Annu Rev Biochem 1999, **68**:965-1014.

[150] Johnston JA, Wang LM, Hanson EP, Sun XJ, White MF, Oakes SA, Pierce JH, O'Shea JJ: **Interleukins 2, 4, 7, and 15 stimulate tyrosine phosphorylation of insulin receptor substrates 1 and 2 in T cells. Potential role of JAK kinases.** J Biol Chem 1995, **270**:28527-30.

[151] Chen Z, Laurence A, Kanno Y, Pacher-Zavisin M, Zhu BM, Tato C, Yoshimura A, Hennighausen L, O'Shea JJ: **Selective regulatory function of Socs3 in the formation of IL-17-secreting T cells.** Proc Natl Acad Sci U S A 2006, **103**:8137-42.

[152] Lin JX, Migone TS, Tsang M, Friedmann M, Weatherbee JA, Zhou L, Yamauchi A, Bloom ET, Mietz J, John S: **The role of shared receptor motifs and common Stat**

proteins in the generation of cytokine pleiotropy and redundancy by IL-2, IL-4, IL-7, IL-13, and IL-15. Immunity 1995, **2**:331-9.

[153] Fujii H, Nakagawa Y, Schindler U, Kawahara A, Mori H, Gouilleux F, Groner B, Ihle JN, Minami Y, Miyazaki T: **Activation of Stat5 by interleukin 2 requires a carboxyl-terminal region of the interleukin 2 receptor beta chain but is not essential for the proliferative signal transmission.** Proc Natl Acad Sci U S A 1995, **92**:5482-6.

[154] Oaks JA, Knowles WJ, Cain GD: **A simple method of obtaining an enriched fraction of tegumental brush border from Hymenolepis diminuta.** J Parasitol 1977, **63**:476-85.

[155] Evans GA, Goldsmith MA, Johnston JA, Xu W, Weiler SR, Erwin R, Howard OM, Abraham RT, O'Shea JJ, Greene WC: **Analysis of interleukin-2-dependent signal transduction through the Shc/Grb2 adapter pathway. Interleukin-2-dependent mitogenesis does not require Shc phosphorylation or receptor association.** J Biol Chem 1995, **270**:28858-63.

[156] Hunt AE, Lali FV, Lord JD, Nelson BH, Miyazaki T, Tracey KJ, Foxwell BM: **Role of interleukin (IL)-2 receptor beta-chain subdomains and Shc in p38 mitogen-activated protein (MAP) kinase and p54 MAP kinase (stress-activated protein Kinase/c-Jun N-terminal kinase) activation. IL-2-driven proliferation is independent of p38 and p54 MAP kinase activation.** J Biol Chem 1999, **274**:7591-7.

[157] Huang J, Sugie K, La Face DM, Altman A, Grey HM: **TCR antagonist peptides induce formation of APC-T cell conjugates and activate a Rac signaling pathway.** Eur J Immunol 2000, **30**:50-8.

[158] Lockyer HM, Tran E, Nelson BH: **STAT5 is essential for Akt/p70S6 kinase activity during IL-2-induced lymphocyte proliferation.** J Immunol 2007, **179**:5301-8.

[159] Horak ID, Gress RE, Lucas PJ, Horak EM, Waldmann TA, Bolen JB: **T-lymphocyte interleukin 2-dependent tyrosine protein kinase signal transduction involves the activation of p56lck** Proc Natl Acad Sci U S A 1991, **88**:1996-2000.

[160] Minami Y, Kono T, Yamada K, Kobayashi N, Kawahara A, Perlmutter RM, Taniguchi T: **Association of p56lck with IL-2 receptor beta chain is critical for the IL-2-induced activation of p56lck** EMBO J 1993 **12**:759-68.

[161] Hatakeyama M, Kono T, Kobayashi N, Kawahara A, Levin SD, Perlmutter RM, Taniguchi T: **Interaction of the IL-2 receptor with the src-family kinase p56lck: identification of novel intermolecular association** Science 1991, **252**:1523-8.

[162] Lindquist JA, Simeoni L, Schraven B: **Transmembrane adapters: attractants for cytoplasmic effectors.** Immunol Rev 2003, **191**:165-82.

[163] Liou J, Kiefer F, Dang A, Hashimoto A, Cobb MH, Kurosaki T, Weiss A: **HPK1 is activated by lymphocyte antigen receptors and negatively regulates AP-1.** Immunity 2000, **12**:399-408.

[164] Qu X, Kawauchi-Kamata K, Miah SM, Hatani T, Yamamura H, Sada K: **Tyrosine phosphorylation of adaptor protein 3BP2 induces T cell receptor-mediated activation of transcription factor.** Biochemistry 2005, **44**:3891-8.

[165] Shan X, Czar MJ, Bunnell SC, Liu P, Liu Y, Schwartzberg PL, Wange RL: **Deficiency of PTEN in Jurkat T cells causes constitutive localization of Itk to the plasma membrane and hyperresponsiveness to CD3 stimulation.** Mol Cell Biol 2000, **20**:6945-57.

[166] Zamoyska R, Basson A, Filby A, Legname G, Lovatt M, Seddon B: **The influence of the src-family kinases, Lck and Fyn, on T cell differentiation, survival and activation.** Immunol Rev 2003, **191**:107-18.

[167] Okkenhaug K, Vanhaesebroeck B: **PI3K in lymphocyte development, differentiation and activation.** Nat Rev Immunol 2003, **3**:317-30.

[168] Reif K, Burgering BM, Cantrell DA: **Phosphatidylinositol 3-kinase links the interleukin-2 receptor to protein kinase B and p70 S6 kinase.** J Biol Chem 1997, **272**:14426-33.

[169] Marks RE, Ho AW, Rivas F, Marshall E, Janardhan S, Gajewski TF: **Differential Ras signaling via the antigen receptor and IL-2 receptor in primary T lymphocytes.** Biochem Biophys Res Commun 2003, **312**:691-6.

[170] Bessoles S, Fouret F, Dudal S, Besra GS, Sanchez F, Lafont V: **IL-2 triggers specific signaling pathways in human NKT cells leading to the production of pro- and anti-inflammatory cytokines.** J Leukoc Biol 2008, **84**:224-33.

[171] Davis RJ: **Signal transduction by the JNK group of MAP kinases.** Cell 2000, **103**:239-52.

[172] Yan M, Dai T, Deak JC, Kyriakis JM, Zon LI, Woodgett JR, Templeton DJ: **Activation of stress-activated protein kinase by MEKK1 phosphorylation of its activator SEK1.** Nature , **372**:798-800.

[173] Guan Z, Buckman SY, Pentland AP, Templeton DJ, Morrison AR: **Induction of cyclooxygenase-2 by the activated MEKK1 –¿ SEK1/MKK4 –¿ p38 mitogen-activated protein kinase pathway.** J Biol Chem 1998, **273**:12901-8.

[174] Dérijard B, Raingeaud J, Barrett T, Wu IH, Han J, Ulevitch RJ, Davis RJ: **Independent human MAP-kinase signal transduction pathways defined by MEK and MKK isoforms.** Science 1995, **267**:682-5.

[175] Nourse J, Firpo E, Flanagan WM, Coats S, Polyak K, Lee MH, Massague J, Crabtree GR, Roberts JM: **Interleukin-2-mediated elimination of the p27Kip1 cyclin-dependent kinase inhibitor prevented by rapamycin.** Nature 1994, **372**:570-3.

[176] Calvo V, Crews CM, Vik TA, Bierer BE: **Interleukin 2 stimulation of p70 S6 kinase activity is inhibited by the immunosuppressant rapamycin.** Proc Natl Acad Sci U S A 1992, **89**:7571-5.

[177] Terada N, Lucas JJ, Szepesi A, Franklin RA, Takase K, Gelfand EW: **Rapamycin inhibits the phosphorylation of p70 S6 kinase in IL-2 and mitogen-activated human T cells.** Biochem Biophys Res Commun 1992, **186**:1315-21.

[178] Kuo CJ, Chung J, Fiorentino DF, Flanagan WM, Blenis J, Crabtree GR: **Rapamycin selectively inhibits interleukin-2 activation of p70 S6 kinase.** Nature 1992, **358**:70-3.

[179] Brennan P, Babbage JW, Thomas G, Cantrell D: **p70(s6k) integrates phosphatidylinositol 3-kinase and rapamycin-regulated signals for E2F regulation in T lymphocytes.** Mol Cell Biol 1999, **19**:4729-38.

[180] Lafont V, Astoul E, Laurence A, Liautard J, Cantrell D: **The T cell antigen receptor activates phosphatidylinositol 3-kinase-regulated serine kinases protein kinase B and ribosomal S6 kinase 1.** FEBS Lett 2000, **486**:38-42.

[181] Pullen N, Dennis PB, Andjelkovic M, Dufner A, Kozma SC, Hemmings BA, Thomas G: **Phosphorylation and activation of p70s6k by PDK1.** Science 1998, **279**:707-10.

[182] Hinton HJ, Alessi DR, Cantrell DA: **The serine kinase phosphoinositide-dependent kinase 1 (PDK1) regulates T cell development.** Nat Immunol 2004, **5**:539-45.

[183] Alessi DR, James SR, Downes CP, Holmes AB, Gaffney PR, Reese CB, Cohen P: **Characterization of a 3-phosphoinositide-dependent protein kinase which phosphorylates and activates protein kinase Balpha.** Curr Biol 1997, **7**:261-9.

[184] Anderson KE, Coadwell J, Stephens LR, Hawkins PT: **Translocation of PDK-1 to the plasma membrane is important in allowing PDK-1 to activate protein kinase B.** Curr Biol 1998, **8**:684-91.

[185] Ciprés A, Carrasco S, Merino E, Díaz E, Krishna UM, Falck JR, Martínez-A C, Mérida I: **Regulation of diacylglycerol kinase alpha by phosphoinositide 3-kinase lipid products.** J Biol Chem 2003, **278**:35629-35.

[186] Mora A, Komander D, van Aalten DM, Alessi DR: **PDK1, the master regulator of AGC kinase signal transduction.** Semin Cell Dev Biol 2004, **15**:161-70.

[187] Rameh LE, Cantley LC: **The role of phosphoinositide 3-kinase lipid products in cell function.** J Biol Chem 1999, **274**:8347-50.

[188] Okkenhaug K, Bilancio A, Emery JL, Vanhaesebroeck B: **Phosphoinositide 3-kinase in T cell activation and survival.** Biochem Soc Trans 2004, **32**:332-5.

[189] Crawley JB, Rawlinson L, Lali FV, Page TH, Saklatvala J, Foxwell BM: **T cell proliferation in response to interleukins 2 and 7 requires p38MAP kinase activation.** J Biol Chem 1997, **272**:15023-7.

[190] Nguyen T, Wang R, Russell JH: **IL-12 enhances IL-2 function by inducing CD25 expression through a p38 mitogen-activated protein kinase pathway.** Eur J Immunol 2000, **30**:1445-52.

[191] Gómez J, García A, R-Borlado L, Bonay P, Martínez-A C, Silva A, Fresno M, Carrera AC, Eicher-Streiber C, Rebollo A: **IL-2 signaling controls actin organization through Rho-like protein family, phosphatidylinositol 3-kinase, and protein kinase C-zeta.** J Immunol 1997, **158**:1516-22.

[192] Hanada M, Feng J, Hemmings BA: **Structure, regulation and function of PKB/AKT–a major therapeutic target.** Biochim Biophys Acta 2004, **1697**:3-16.

[193] Bierer BE, Mattila PS, Standaert RF, Herzenberg LA, Burakoff SJ, Crabtree G, Schreiber SL: **Two distinct signal transmission pathways in T lymphocytes are inhibited by complexes formed between an immunophilin and either FK506 or rapamycin.** Proc Natl Acad Sci U S A 1990, **87**:9231-5.

[194] Terada N, Lucas JJ, Szepesi A, Franklin RA, Domenico J, Gelfand EW: **Rapamycin blocks cell cycle progression of activated T cells prior to events characteristic of the middle to late G1 phase of the cycle.** J Cell Physiol 1993, **154**:7-15.

[195] Brunn GJ, Williams J, Sabers C, Wiederrecht G, Lawrence JC, Abraham RT: **Direct inhibition of the signaling functions of the mammalian target of rapamycin by the phosphoinositide 3-kinase inhibitors, wortmannin and LY294002.** EMBO J 1996, **15**:5256-67.

[196] Zheng Y, Collins SL, Lutz MA, Allen AN, Kole TP, Zarek PE, Powell JD: **A role for mammalian target of rapamycin in regulating T cell activation versus anergy.** J Immunol 2007, **178**:2163-70.

[197] Duré M, Macian F: **IL-2 signaling prevents T cell anergy by inhibiting the expression of anergy-inducing genes.** Mol Immunol 2009, **46**:999-1006.

[198] Flores I, Jones DR, Mérida I: **Changes in the balance between mitogenic and antimitogenic lipid second messengers during proliferation, cell arrest, and apoptosis in T-lymphocytes.** FASEB J 2000, **14**:1873-5.

[199] Teramoto H, Coso OA, Miyata H, Igishi T, Miki T, Gutkind JS: **Signaling from the small GTP-binding proteins Rac1 and Cdc42 to the c-Jun N-terminal kinase/stress-activated protein kinase pathway. A role for mixed lineage kinase 3/protein-tyrosine kinase 1, a novel member of the mixed lineage kinase family.** J Biol Chem 1996, **271**:27225-8.

[200] Minden A, Lin A, Claret FX, Abo A, Karin M: **Selective activation of the JNK signaling cascade and c-Jun transcriptional activity by the small GTPases Rac and Cdc42Hs.** Cell 1995, **81**:1147-57.

[201] Zakaria S, Gomez TS, Savoy DN, McAdam S, Turner M, Abraham RT, Billadeau DD: **Differential regulation of TCR-mediated gene transcription by Vav family members.** J Exp Med 2004, **199**:429-34.

[202] Franklin RA, Tordai A, Patel H, Gardner AM, Johnson GL, Gelfand EW: **Ligation of the T cell receptor complex results in activation of the Ras/Raf-1/MEK/MAPK cascade in human T lymphocytes.** J Clin Invest 1994, **93**:2134-40.

[203] Burns LA, Karnitz LM, Sutor SL, Abraham RT: **Interleukin-2-induced tyrosine phosphorylation of p52shc in T lymphocytes.** J Biol Chem 1993, **268**:17659-61.

[204] Zhu X, Suen KL, Barbacid M, Bolen JB, Fargnoli J: **Interleukin-2-induced tyrosine phosphorylation of Shc proteins correlates with factor-dependent T cell proliferation.** J Biol Chem 1994, **269**:5518-22.

[205] Delespine-Carmagnat M, Bouvier G, Bertoglio J: **Association of STAT1, STAT3 and STAT5 proteins with the IL-2 receptor involves different subdomains of the IL-2 receptor beta chain.** Eur J Immunol 2000, **30**:59-68.

[206] Kallies A, Hawkins ED, Belz GT, Metcalf D, Hommel M, Corcoran LM, Hodgkin PD, Nutt SL: **Transcriptional repressor Blimp-1 is essential for T cell homeostasis and self-tolerance.** Nat Immunol 2006, **7**:466-74.

[207] Martins GA, Cimmino L, Shapiro-Shelef M, Szabolcs M, Herron A, Magnusdottir E, Calame K: **Transcriptional repressor Blimp-1 regulates T cell homeostasis and function.** Nat Immunol 2006, **7**:457-65.

[208] Sanjuán MA, Jones DR, Izquierdo M, Mérida I: **Role of diacylglycerol kinase alpha in the attenuation of receptor signaling.** J Cell Biol 2001, **153**:207-20.

[209] Hehner SP, Hofmann TG, Dienz O, Droge W, Schmitz ML: **Tyrosine-phosphorylated Vav1 as a point of integration for T-cell receptor- and CD28-mediated activation of JNK, p38, and interleukin-2 transcription.** J Biol Chem 2000, **275**:18160-71.
