# Supplementary material for: Integrating Signals from the T-Cell Receptor and the Interleukin-2 Receptor
Source: PLoS Comput Biol. 2011 Aug 4;7(8):e1002121. doi: 10.1371/journal.pcbi.1002121 (PMC3150289; doi:10.1371/journal.pcbi.1002121)
Supplement: Table S3 — Activation states of the merged network upon different stimuli. Lists the state vectors for the merged network with TCR and IL-2R stimulation alone or in combination. (PDF) [file pcbi.1002121.s014.pdf]

Table S3

Activation states of the merged TCR + IL2 network upon different stimuli. TCR=stimulation of the TCR without IL-2 stimulation at early time points (tau=1); IL2=stimulation with IL-2 without TCR stimulation at early time points (tau=1); TCR+IL2=simultaneous stimlation of both the TCR and the IL2R at early time points (tau=1); IL2pre=stimulation with IL-2 taking into account late interactions (tau=2) without determining whether TCR is triggered or not, under this condition not every node has a uniquely determined activation state (ND=not determined); IL2pre-woTCR= IL2pre and setting TCR stimulation to zero (TCRLIG=0); TCR-with-IL2pre=IL2pre with subsequent TCR stimulation (TCRLIG=1)

	no-stimulus	TCR	IL2	TCR +IL2	IL2pre	IL2pre-woTCR	TCR-with-IL2pre
A20:	0	0	0	0	0	0	0
ABL:	0	1	1	1	1	1	1
Aiolos:	0	1	1	1	1	1	1
AKAP79:	0	0	0	0	0	0	0
AP1:	0	1	1	1	1	1	1
b-catenin:	0	1	1	1	0	0	0
BAD:	1	0	0	0	1	1	1
BCL10:	1	1	1	1	1	1	1
Bcl2:	0	1	1	1	1	1	1
BCLXL:	0	1	1	1	0	0	0
Blimp-1:	0	0	0	0	0	0	0
BM:	0	1	0	1	0	0	1
c-myc:	0	0	1	1	1	1	1
c-RAF:	0	0	0	0	1	1	1
CA:	0	1	0	0	0	0	0
CABIN1:	1	0	1	1	1	1	1
calcineurin:	0	1	0	0	0	0	0
CaM:	0	1	0	0	0	0	0
CAMK2:	0	1	0	0	0	0	0
CAMK4:	0	1	0	0	0	0	0
cAMP:	0	0	0	0	0	0	0
CARD11:	1	1	1	1	1	1	1
CASPASE-8:	0	1	0	1	0	0	1
Cbl:Grb2:	0	0	0	0	1	1	1
Cbl:Grb2:Shc:	0	0	0	0	0	0	0
CBLB:	0	0	0	0	0	0	0
CBM:	0	1	0	1	0	0	1
CCBL:	0	0	0	0	1	1	1
CCBLP1:	0	0	0	0	0	0	0
CCBLP2:	0	0	0	0	1	1	1
CCBLR:	1	1	1	1	1	1	1
CD28:	0	0	0	0	0	0	0
CD4:	0	0	0	0	0	0	0
CD45:	1	1	1	1	1	1	1
CDC42:	0	0	0	0	0	0	0
cFLIP:	1	1	1	1	1	1	1
cFLIP-p22:	0	1	0	1	0	0	1
cFLIP-p43:	0	1	0	1	0	0	1
CRE:	0	1	1	1	0	0	0
CREB:	0	1	1	1	0	0	0
CrkL:	0	0	1	1	1	1	1
CrkL:p85:	0	0	1	1	0	0	0
CSK:	0	0	1	0	ND	1	0

	no-stimulus	TCR	IL2	TCR +IL2	IL2pre	IL2pre-woTCR	TCR-with-IL2pre
Csp1:	0	0	0	0	0	0	0
CYC1:	0	1	1	1	0	0	0
cyclin A:	0	1	1	1	0	0	0
cyclin D3:	0	1	1	1	1	1	1
cyclin/cdk:	0	1	1	1	0	0	0
DAG:	0	1	0	0	0	0	0
DAG (PLA):	0	0	0	0	0	0	0
DAG (PLD):	0	0	1	1	1	1	1
DGK:	0	0	0	0	1	1	1
DGKa:	0	0	0	0	1	1	1
E2F:	0	1	1	1	0	0	0
ERK:	0	1	1	1	0	0	0
FKHR:	1	0	0	0	1	1	1
FOS:	0	1	1	1	1	1	1
FYN:	0	1	1	1	1	1	1
GAB2:	0	0	1	1	0	0	0
GADD45:	0	0	0	0	0	0	0
GADS:	0	1	1	1	1	1	1
GAP:	0	0	0	0	0	0	0
GRB2:	0	1	1	1	1	1	1
GSK3:	1	0	0	0	1	1	1
HPK1:	0	1	1	1	1	1	1
IKB:	1	0	1	1	1	1	1
IKKAB:	0	1	0	0	0	0	0
IKKG:	0	1	0	0	0	0	0
IL2:	0	0	1	1	1	1	1
IL2Ra:	1	1	1	1	1	1	1
IL2Rabg:	0	0	1	1	1	1	1
IL2Rb:	1	1	1	1	1	1	1
IL2Rbg:	1	1	1	1	1	1	1
IL2Rgc:	1	1	1	1	1	1	1
IP3:	0	1	0	0	0	0	0
IRS:	0	0	1	1	0	0	0
ITK:	0	1	0	0	0	0	0
JAK1:	0	0	1	1	0	0	0
JAK3:	0	0	1	1	0	0	0
JNK:	0	1	1	1	1	1	1
JUN:	0	1	1	1	1	1	1
LAT:	0	1	1	1	1	1	1
LCK:	0	1	1	1	1	1	1
LCKP1:	0	0	0	0	0	0	0
LCKP2:	0	1	0	1	0	0	0
LCKR:	1	1	1	1	1	1	1
MALT1:	1	1	1	1	1	1	1
MEK:	0	1	1	1	1	1	1
MEKK1:	0	1	1	1	1	1	1
MKK4:	0	1	1	1	1	1	1
MLK3:	0	1	1	1	1	1	1
mTOR:	0	1	1	1	0	0	0
NFAT:	0	1	0	0	0	0	0
NFKB:	0	1	0	0	0	0	0
nPKC:	0	1	1	1	1	1	1

	no-stimulus	TCR	IL2	TCR +IL2	IL2pre	IL2pre-woTCR	TCR-with-IL2pre
P21Cip1:	1	0	0	0	1	1	1
P27Kip:	1	0	0	0	1	1	1
P38:	0	1	1	1	1	1	1
P70S6K:	0	1	1	1	0	0	0
PA:	0	0	0	0	1	1	1
PAG:	0	0	1	0	ND	1	0
pCbl:pCrkL:	0	0	0	0	1	1	1
PDK1:	0	1	1	1	0	0	0
pGab2:CrkL:	0	0	1	1	0	0	0
PI3K:	0	1	1	1	0	0	0
pIL2R:	0	0	0	0	0	0	0
PIP3:	0	1	1	1	0	0	0
PKB:	0	1	1	1	0	0	0
PKCTH:	0	1	0	0	0	0	0
PKCz:	0	1	1	1	0	0	0
PLA:	0	0	0	0	0	0	0
PLCGA:	0	1	0	0	0	0	0
PLCGB:	0	1	1	1	1	1	1
PLD:	0	0	1	1	1	1	1
pRB:	0	1	1	1	0	0	0
PTEN:	0	0	0	0	0	0	0
RAC1P1:	0	1	0	1	ND	0	1
RAC1P2:	0	1	0	1	ND	0	1
RAC1R:	1	1	1	1	1	1	1
RAF:	0	1	1	1	1	1	1
RAS:	0	1	1	1	1	1	1
RASGRP:	0	1	0	0	0	0	0
RIP1:	0	1	0	1	0	0	1
RIP2:	0	1	0	1	0	0	1
RLK:	0	0	0	0	0	0	0
RSK:	0	1	1	1	0	0	0
S6:	0	1	1	1	0	0	0
SH3BP2:	0	1	0	1	ND	0	1
Shc:	0	0	0	0	0	0	0
SHIP1:	0	0	0	0	0	0	0
SHP1:	0	0	0	0	1	1	1
SHP2:	0	0	1	1	0	0	0
SLP76:	0	1	0	0	ND	0	1
SOCS-1:	0	0	0	0	0	0	0
SOCS-3:	0	0	0	0	0	0	0
SOS:	0	1	1	1	1	1	1
SRE:	0	1	0	1	ND	0	1
STAT3:	0	1	1	1	1	1	1
STAT5:	0	1	1	1	1	1	1
Syk:	0	0	1	1	0	0	0
TCRB:	0	1	0	1	ND	0	1
TCRLIG:	0	1	0	1	ND	0	1
TCRP:	0	1	0	1	ND	0	1
TRAF2:	0	1	0	1	0	0	1
TRAF6:	0	1	0	1	0	0	1
VAV1:	0	1	0	1	ND	0	1
VAV3:	0	1	0	1	ND	0	1
X:	0	0	0	0	0	0	0
ZAP70:	0	1	0	1	ND	0	1
